# About feasibility of SpaceX's human exploration Mars mission scenario with Starship

**DOI:** 10.1038/s41598-024-54012-0

**Published:** 2024-05-23

**Authors:** Volker Maiwald, Mika Bauerfeind, Svenja Fälker, Bjarne Westphal, Christian Bach

**Affiliations:** 1 German Aerospace Center (DLR), Institute of Space Systems, Bremen, Germany; 2 Faculty of Production Engineering, University of Bremen, Bremen, Germany; 3 Chair of Space Systems, Technical University Dresden, Dresden, Germany; 4 Technical University Braunschweig, Braunschweig, Germany

**Keywords:** Mission feasibility, Human spaceflight, Future space missions, Mars, Aerospace engineering, Mechanical engineering

## Abstract

After decades where human spaceflight missions have been reserved to low Earth orbit, recent years have seen mission proposals and even implemented plans, e.g. with the mission Artemis I, for returning to the lunar surface. SpaceX has published over various media (e.g., its official website, conference presentations, user manual) conceptual information for its reusable Starship to enable human exploration missions to the Martian surface by the end of the decade. The technological and human challenges associated with these plans are daunting. Such a mission at that distance would require excellent system reliability and in-situ-resource utilization on a grand scale, e.g. to produce propellant. The plans contain little details however and have not yet been reviewed concerning their feasibility. In this paper we show significant technological gaps in these plans. Based on estimates and extrapolated data, a mass model as needed to fulfill SpaceX’s plans could not be reproduced and the subsequent trajectory optimization showed that the current plans do not yield a return flight opportunity, due to a too large system mass. Furthermore, significant gaps exist in relevant technologies, e.g. power supply for the Martian surface. It is unlikely that these gaps can be closed until the end of the decade. We recommend several remedies, e.g. stronger international participation to distribute technology development and thus improve feasibility. Overall, with the limited information published by SpaceX about its system and mission scenario and extrapolation from us to fill information gaps, we were not able to find a feasible Mars mission scenario using Starship, even when assuming optimal conditions such as 100% recovery rate of crew consumables during flight.

## Introduction

In 1952 Wernher von Braun published *The Mars Project*^1^, the first feasibility study and technical, non-fictional scenario for a human mission to Mars, describing how a crew of 70 people would reach Mars and stay on its surface for more than a year. The *Martian Piloted Complex* was a soviet study for a Mars mission, drafted by Mikhail Tikhonravov in 1956 over the course of several years. Using a spacecraft that would be assembled with 25 N1 rockets, the never successful soviet launch vehicle for lunar missions, a six-person crew would conduct a 900-day mission to Mars.

Numerous more studies for human Mars missions were conducted by different actors, national, commercial and academic over the course of the past decades. No study ever led to an actual mission. It is a truism that technologically, economically, psychologically and physiologically any other human explorative endeavor pales before a crewed mission to Mars. Life-support systems (LSS) capable of ensuring human survival for several years, do not yet exist with sufficient reliability. In-situ resource utilization (ISRU), e.g. to supply missions with fuel, water and oxygen on the Martian surface, has only been tested under lab conditions. Pro-longed stay in micro-gravity negatively affects the health of the human crew and countermeasures have to be developed.

SpaceX Starship is a spacecraft in development, which is intended to be used for the first human landing on the lunar surface in more than five decades and according to SpaceX’s owner, Elon Musk, will also be the enabler for the first human Mars mission^2^. The most recent information about a possible launch date for such a human exploration mission has been a Tweet from 2022, which mentions that his plan envisions such a human mission in 2029^2^. The last excursion beyond LEO has happened 50 years ago and the currently planned lunar missions have not yet reached an operational phase with crew. Artemis has conducted a lunar orbital mission, Gateway is currently still in development. With that status, plans for a human Mars mission, especially in that time frame warrant close inspection.

Artemis’ Orion vehicle and Gateway use similar systems, e.g. concerning life-support, partially developed from the International Space Station (ISS)^3^, forming an integrated path of different mission scenarios, lunar and Martian exploration. Gateway’s early configuration will consist of the Power and Propulsion Element (PPE), which has been contracted to Maxar, and the Habitation and Logistics Outpost (HALO)^4^. Currently the launch of both modules is planned for 2025^5^. The PPE equips Gateway with a 60 kW solar array as well as an electrical propulsion system for orbit control^6^. It will also act as communication relay for communication between Moon and Earth and link Gateway with Earth^4^.

Published mission scenarios for Starship rely heavily on refueling, including on the Martian surface using ISRU to supply the fuel needed for the return flight to Earth^7^. Reliance on such technology for the return flight mandates a significant reliability to avoid stranding the crew on Mars. Setting up such an infrastructure on the Martian surface poses an own challenge, as such infrastructure exceeds anything ever transported beyond LEO. Other options, e.g. using refueling depots are not published by SpaceX. These are some challenges which have to be overcome for a successful Mars mission utilizing Starship, specifically—in addition to those previously mentioned general challenges.

### Motivation, goals and approach

The prominence of Starship in current mission plans, e.g. as Human Landing System for *Artemis III* on the lunar surface, and its intended role within Mars mission plans warrant an analysis concerning the feasibility of such Mars missions. This goes along with establishing an understanding of mission efficiency sensitivity and effectiveness concerning parameters such as launch date. The goal of the work is a profound understanding of current gaps in the mission architecture and system design and to provide recommendations to remedy these gaps. The basic premise is to assume how a mission based on the scenario painted by SpaceX would work out and if it is plausible. This requires a compilation of statements and data about this scenario and careful analysis—where necessary with extrapolation—of this data, including e.g. trajectory calculations. Based on these a statement about the plausibility, feasibility of the mission as drawn out by SpaceX for its Starship, can be and is made.

First, data is compiled about the current mission scenario, where necessary data is extrapolated and assumptions are used to fill gaps in the data. This compiled baseline is then weighed against the requirements of the Mars mission as provided by the current scenario^7^. A mass budget is established and from that a trajectory analysis used to determine e.g. propellant needs to be covered by ISRU on the Martian surface. Subsequently, the feasibility is analyzed and discussed e.g. considering the technology readiness level of the required technologies, available payload mass or $\Delta v$ and thus propellant mass required. The major questions to be answered are:

- Is the mass information provided by SpaceX plausible?
- Is the propellant mass as given by SpaceX plausible for the given mission scenario(s)?
- Is the time of flight (ToF) aimed at by SpaceX plausible?
- What is the energy need for ISRU activities on the Martian surface and how can it be satisfied?

Overall, gaps shall be identified and discussed, how these gaps can be closed to improve the feasibility of a Mars mission using SpaceX Starship. The feasibility of Starship is evaluated based on the numbers given in Table 1, as published by SpaceX. More context on these numbers is given in the following sections of this work.

**Table 1:** Summary of numbers provided by SpaceX concerning its Starship mission scenario. Information about ISRU technologies is not provided as SpaceX does not provide any.

Parameter	Value	Unit	Source
System mass incl. 20% margin	100	MT	^8^
Cryogenic methane storage on Starship	1200	MT	^9^
Cryogenic methane storage on Super Heavy	3600	MT	^10^
Propellant tank mass Super Heavy	80	MT	^10^
Payload mass	100	MT	^11^
Maximum permissible perigee velocity on Mars landing trajectory	7500	$\mathrm{m }{{\text{s}}}^{-1}$	^7^
Maximum permissible perigee velocity on Earth landing trajectory	12,500	$\mathrm{m }{{\text{s}}}^{-1}$	^12^

### Paper outline

Section "Analysis method" describes the method used for the analysis of feasibility for SpaceX Starship. Subsequently, in Section "Results" the results are shown, first describing the mass budget as a basic representation of Starship for further analysis. Special emphasis is given on the trajectory analysis and ISRU. The latter is an essential part of the mission design, a key element for feasibility as it reduces the amount of fuel (and potentially other materials) to be taken along by Starship to Mars. In Section "Discussion" the results are discussed, evaluating the plausibility of the current scenario and certain key aspects, e.g. ISRU providing fuel, technology readiness (for the envisioned time frame of 2029) and adding recommendations for keeping that schedule and improving mission feasibility. Finally, the conclusion summarizes the findings of this work.

## Analysis method

First, a literature review is conducted to compile available data on Starship’s system and mission design. The found information is filtered, based on recency and reliability. From this a baseline design for Starship and the mission scenario is selected, which will be used for the subsequent analysis. The mass budget is set up using the European Space Agency’s (ESA) margin philosophy for feasibility studies of science missions^13^. Specifically, this means that an overall 20% margin is assumed for the system mass—this accounts for additional, currently unforeseen elements, e.g. additional batteries, tank size changes. On component level, margins can be described with three levels:

- 5%—off the shelf items, i.e. items are essentially unchanged and the margin covers only additional bolts, screws and similar elements
- 10%—to be modified, i.e. items with heritage are used as baseline, but need changes for new functionalities or performance improvement
- 20%—to be developed, i.e. the estimate is quite inaccurate as the element is to be developed from scratch, even though some comparable element might exist to derive parameters from

The process is partly iterative as the data is interdependent, e.g. the amount of propellant to be generated depends on the amount of propellant needed for the transfer, which is a function of the system mass, but also e.g. launch date.

This philosophy has been picked for reasons of familiarity by the authors and due to the unprecedented nature of the proposed Mars mission. It is not reflecting the assumption that that design philosophy will be applied by SpaceX, but merely serves our own estimations.

### Starship system and payload masses

To review the feasibility of Starship’s Mars mission as proposed by SpaceX, all relevant data for the spacecraft were compiled first. This data was obtained from publications by SpaceX (e.g.^7,14, 15^) or about SpaceX (e.g.^9,10, 16^) where the former were not available. In case of contradicting information, the most recent one was selected, to consider possible updates on the design. Where no information was available about Starship, data was extrapolated from existing systems, e.g. based on Orion technology. The system design also includes ISRU-technology.

Since the topic of Starship is still new and subject to recurring changes, the search was conducted purely via digital sources, including video interviews of Elon Musk, e.g.^10^, or presentations, e.g.^17^. In the search for further components and technologies for Starship, NASA and other space companies’ references were consulted for information on existing elements and those in development. In addition, further requirements have been set for the Starship that still have to be fulfilled.

Subsequently, the following steps were taken:

1. Definition of the subsystems,
2. Set-up of the respective system designs,
3. Estimation of mass and power budgets where possible,
4. Estimation of mass related to crew and consumables, assuming the best case of 100% recoverable consumables.

With this compiled system design, the mission feasibility concerning the given mission scenario has been analysed and evaluated subsequently. For the feasibility analysis the most relevant key figures have been identified, which can be addressed with the available information. Since Orion is currently the most analogue spacecraft based on its exploration mission purpose, elements, which could not be determined in mass in any other manner, were extrapolated based on mass information of Orion, as compiled in Table 2 (based on^18^). Another option as basis for extrapolation has been Lunar Gateway. However, during preparation of this work, specified data about Gateway has been scarce and not available in the detail needed for the intended extrapolation. Furthermore, the mission purpose of Orion—designed as human exploration vehicle for missions beyond LEO, including Mars^19^ (even though not with using only the capsule)—is more akin to that of Starship than the mission of Lunar Gateway which is intended to be an outpost in lunar orbit.

**Table 2:** Mass summary for Orion, based on information given in^18^.

Subsystem	Mass contribution by CM [kg]	Mass contribution by SM [kg]	Sum [kg]	Ratio subsystem of dry mass
Structure	1883	819	2702	22.72%
Protection (e.g. thermal, debris protection)	894	167	1061	8.92%
Propulsion	413	1423	1836	15.44%
Power	819	417	1236	10.39%
Avionics (communication, data handling, command, harness, guidance and navigation)	435	117	552	4.64%
Environment (life-support, thermal control and crew accomodation)	1091	98	1189	10%
Other (e.g. docking mechanisms, hatches, parachutes, landing airbags)	1159	290	1449	12.18%
Non-cargo (crew, crew provisions, propellant residuals)	821	579	1400	11.77%
Total dry mass			**11,892**	
Margin (20% of dry mass)	–	–	2378	
Cargo	100	0	100	
Non-propellant (e.g. Oxygen, Nitrogen, potable water)	367	0	367	
Propellant	184	9071	9255	
**Total system mass**	–	–	**23,992**	

Partially used for extrapolating missing information about Starship.

Significant values are in bold.

### Trajectory analysis

For the trajectory analysis, baseline data is taken from literature to establish a mission scenario. This mission scenario is used to calculate fitting trajectories, searching for minimum Δ*v* mission opportunities and those with minimum Time of Flight (ToF) to find those fitting SpaceX published flight times with goals of 80 days and even 30 days^20^. All calculations were conducted with parameter values for standard parameters as given in Table 3.

**Table 3:** Parameter values as used for trajectory calculations within this work.

Parameter	Value
Standard gravitational acceleration *g*_0_	9*.*806 65 m s^*−*2^
Gravitational parameter of Mars *µ*_M_	4*.*282 837 *·* 10^4^ km^3^ s^*−*2^
Gravitational parameter of Earth *µ*_E_	3*.*986 004 418 *·* 10^5^ km^3^ s^*−*2^
Gravitational parameter of the Sun *µ*_S_	1*.*327 124 400 18 *·* 10^11^ km^3^ s^*−*2^

In general, the trajectory analysis of an interplanetary mission can be broken down to the problem of given departure and arrival position vectors, as well as the ToF between them. Finding the trajectory between Earth and Mars has been accomplished using a Lambert solver based on an algorithm developed by Battin and Vaughan^21^ and was implemented in Matlab according to the pseudocode provided by Vallado and McClain^22^. It uses the position vectors of Earth (on departure date) and Mars (on departure date+ToF). The position vectors are modeled with the use of mean orbital elements.

Mean orbital elements are time dependent, linear functions that describe the run of the six Keplerian elements over a long time-interval. The values that describe the functions have been compiled and presented by Seidelmann^23^. The Lambert solver then returns the Δ*v*-values for the necessary maneuvers at Earth and Mars to connect the two planets with an elliptic transfer trajectory. This does not account for the influence of Earth’s and Mars’ gravitation on Starship, therefore the values are adapted with the use of patched conics. Since SpaceX plans to refuel the Starships in LEO, the orbital altitude must be chosen high enough to not deorbit due to the atmospheric drag. An orbital radius of 6878 km and hence an altitude of 500 km has thus been selected. In order to travel on the elliptic trajectory, Starship must leave LEO on a hyperbolic trajectory in relation to Earth. At the perigee of the hyperbola, Starship must have the velocity ${v}_{p,E}$:

1 $$v_{{p,E}} = \sqrt {\frac{{2\mu _{E} }}{{r_{{p,E}} }} + v_{{\infty ,E}}^{2} }$$

where ${v}_{\infty ,E}$ must be equal to the velocity at Earth’s heliocentric position as returned by the Lambert solver, ${\mu }_{E}$ is the gravitational parameter of Earth and ${r}_{p,E}$ is the hyperbola’s pericenter distance at Earth. Therefore, the needed Δ*v-*value of the boost to be performed by Starship at the transfer orbit injection (TOI) maneuver is:

2 $${\Delta v}_{E}=\sqrt{\frac{2{\mu }_{E}}{{r}_{p,E}}+{v}_{\infty ,E}^{2}}-\sqrt{\frac{{\mu }_{E}}{{r}_{p,E}}}$$

During the flight to Mars, interplanetary probes in the past have conducted trajectory correction maneuvers (TCM), with the magnitude of Δ*v* ranging up to 33 m/s for the Pathfinder mission^24^. Considering the relatively low achieved landing accuracy of 30 km^24^, it can be assumed that Starship will need a higher Δ*v*_C_ to ensure precise landing, e.g. close to installed infrastructure. We therefore estimated the total Δ*v* for all TCM during the flight to Mars to be 200 m/s.

When approaching Mars, Starship is travelling on a hyperbolic Keplerian orbit with respect to Mars. It is designed to remove 99% of its kinetic energy purely with aerobraking when the velocity at the perigee of the arrival hyperbola does not exceed 7.5 km/s^25^. In order to be able to use aerobraking, Lu suggests that the perigee should be at an orbital altitude of 129 km^26^. If the perigee velocity is higher, the velocity in excess must be removed by a propulsive maneuver. Therefore, the Δ*v* for the Mars orbit insertion (MOI) maneuver is described by the following equation for a perigee velocity larger than 7.5 km/s:

3 $${\Delta v}_{M}=\sqrt{\frac{2{\mu }_{M}}{{r}_{p,M}}+{v}_{\infty ,M}^{2}}-7.5 \frac{{\text{km}}}{{\text{s}}}$$

Again, ${v}_{\infty ,M}$ must be equal to the velocity at Mars’ heliocentric position as of the results by the Lambert solver. After this maneuver, Starship enters its landing phase. The required Δ*v* for landing is dependent on the payload mass and the graphs by SpaceX show a linear relation between payload mass and Δ*v*^12^. Extraction of the values from the slides gives the following empirical equation to describe the required Δ*v* for landing with respect to the payload mass:

4 $${\Delta v}_{L}=2.088 \frac{{\text{m}}}{{\text{s}}\cdot {\text{t}}}\cdot {m}_{PL}+367.53\frac{{\text{m}}}{{\text{s}}}$$

Now, one can put together the four Δ*v* maneuvers to obtain the total required Δ*v* for a transfer from LEO to the Martian surface. To make the results more robust, Δ*v* margins according to the ESA standards^13^ have been applied to the results.

5 $${\Delta v}_{E\to M}=1.05\cdot \left({\Delta v}_{E}+{\Delta v}_{M}+{\Delta v}_{L}\right)+2{\cdot \Delta v}_{C}$$

As described above, the position vectors depend on the departure date and the ToF, therefore one can form tuples of these two variables and calculate the total required Δ*v* for a transfer to Mars for different dates and ToF. The step size for both variables was chosen to be twelve hours. If the Δ*v* value for a tuple is exceeding the maximum capacity, the value is set to ‘Not a Number’ in the code. Another important figure of merit for evaluating the feasibility of SpaceX’ plans is the maximum payload mass that can be brought to the Martian surface. Since most of the trajectories will not consume all of the Δ*v* available, the payload mass that is carried on these trajectories can be increased. Mathematically, the maximum payload mass is the mass for which the two sides of the following equations equal:

6 $${I}_{sp}\cdot {g}_{0}\cdot {\text{ln}}\left(\frac{{m}_{P}+{m}_{S}+{m}_{PL}}{0.02\cdot {m}_{P}+{m}_{S}+{m}_{PL}}\right)=1.05\cdot \left({\Delta v}_{E}+{\Delta v}_{M}+2.088\frac{{\text{m}}}{{\text{s}}\cdot {\text{t}}}\cdot {m}_{PL}+367.53\frac{{\text{m}}}{{\text{s}}}\right)+2\cdot {v}_{C}$$

This equation cannot be solved analytically and therefore, one must increase the payload mass starting from 100 metric tons (MT) until the right side of the equation is less than 1 m/s lower than the left side. This mass value is then considered to be the maximum.

#### Lambert solver

In order to solve Lambert’s problem for the transfer between Earth and Mars, we implemented a Lambert solver in Matlab. As mentioned before, we used the algorithm provided by Vallado and McClain to solve the Lambert’s equation:

7 $$\Delta t= \sqrt{\frac{{a}^{3}}{{\mu }_{S}}}\left({E}_{2}-{E}_{1}-e\left({\text{sin}}{E}_{1}-{\text{sin}}{E}_{2}\right)\right)$$

where $E$ marks the eccentric anomaly of each planet, respectively, $e$ the eccentricity of the transfer ellipse, $a$ the semi-major axis of the transfer ellipse and ${\mu }_{S}$ the gravitational parameter of the sun. One can re-arrange this equation as shown by Prussing and Conway^27^, which allows the following formulation of Eq. (7):

8 $$\Delta t= \sqrt{\frac{{a}^{3}}{{\mu }_{S}}}\left(\alpha -\beta +\left({\text{sin}}\beta -{\text{sin}}\alpha \right)\right)$$

As $\alpha$ and $\beta$ are both functions dependent only on $a$ and the two position vectors, Eq. (8) allows to directly link the desired time of flight $\Delta t$ and the transfer trajectory. This is the general formulation of the Lambert’s equation and the basic equation the algorithm aims to solve. We implemented a Lambert solver based on the algorithm developed by Battin and Vaughan^21^ and directly mirroring the pseudocode presented by Vallado and McClain^22^. In the loop-section of the code as provided by Vallado and McClain, the stopping condition is defined as: “Until *x* stops changing”. We decided to implement this condition in a while-loop in Matlab that stops when two consecutive values of *x* differ less than ${10}^{-12}$. Inside of the loop, two continued fractions are evaluated, where we decided to use ${c}_{\eta }$ up until *n* = 6 and ${c}_{U}$ up until *n* = 11.

Since the code needs a quantization of the departure date as well as the time of flight, both are evaluated in steps of 24 h. For each possible combination of departure date within a launch opportunity and time of flight, the aforementioned algorithm is evaluated, and the *Δv* is retrieved.

#### Return flight

The return flight was modeled with the same approach as the flight from Earth to Mars, with respect to the Lambert solver and the patched conics. The main, and key, difference is that for the return flight, Starship needs to ascent into a Low Mars Orbit (LMO) by itself. Regarding the *Δv* for such a maneuver, SpaceX does not disclose any information. Therefore, we decided to collect data from different sources and studies and extrapolated values for Starship from this dataset.

We assume that Starship will ascend into a 250 km-altitude, circular orbit. This orbit around Mars has a velocity of 3430 m/s. During the ascent, Starship (or any spacecraft in general) is exposed to losses that decelerate its movement and need to be overcome by further Δv. One distinguishes between gravitational and atmospheric losses, where gravitational losses are a result of the gravitational attraction of Mars and atmospheric losses are a result of drag.

One can express the Δv to reach an LMO can be expressed as follows.

9a $${\Delta v}_{LMO}=\mathrm{3,430 }\frac{{\text{m}}}{{\text{s}}}+ {\Delta v}_{Losses}$$

While the first part of the equation is a constant, the second part is dependent on the spacecraft.

In general, the main figure that influences losses during ascent, is the thrust-to-weight-ration (TWR) of the spacecraft. The higher the TWR is, the faster the spacecraft will ascend, leading to lower gravitational losses since the time spent being exposed to high gravitational forces is shorter. On the other hand, since it reaches higher velocities within the atmosphere, the drag is—and hence the atmospheric losses are—higher. For a lower TWR, the effects apply vice versa.

Before we evaluate the data available, it is important to know the TWR of Starship on Mars. We assume a wet mass of 1310.5 MT for the return flight, what is equivalent to a weight of 4,835,745 N on the Martian surface. The thrust of Starship is given with 1500 tf (tonne-force) by SpaceX^31^. This is equivalent to a thrust of 13,629,975 N. Therefore, the TWR of Starship is 2.819 on the Martian surface.

Detailed information is available for four design studies of a Mars ascent vehicle (MAV). The numbers are presented in the Table 4 table along the subsequently derived values for the respective TWR and losses in Δ*v*. With these, a quadratic fit yields the following equation for the ${\Delta v}_{Losses}$ (y) depending on the TWR (x).

**Table 4:** Properties of the Mars Ascent Vehicle designs from various studies (left), used to calculate their respective TWR (Thrust divided by wet mass times gravitational acceleration of Mars) and Δ*v*_losses_ (= Δ*v* to reach orbit – velocity of target orbit), given on the right side of the table.

Design study	Wet mass [kg]	Thrust [N]	Δ*v* to reach orbit [m/s]	TWR	Δ*v*_losses_ [m/s]
Polsgrove et al.^28^	42,749	300,000	5274	1.902	1844
Trent, Thomas and Rucker^29^	36,827	178,000	5109	1.310	1679
Sopegno et al.^30^ – solid engine	357	5490^1^	4384	4.162	954
Sopegno et al.^30^ – hybrid engine	270	5700	4375	5.723	945

^1^The thrust of the solid engine was not explicitly mentioned, so we assumed a linear relation between the thrust of the solid and hybrid engine. For the hybrid engine, a specific impulse of 298 s was mentioned, for the solid engine, the specific impulse was 287 s. Therefore, thrust for the solid engine is assumed to be 287/298 times the impulse of the hybrid engine.

9b $$y=36.803 {x}^{2}-469.26 x+2382.2$$

Therefore, the delta-v for the losses of Starship can be assumed to be 1352 m/s. This allows us to directly express the needed Δ*v* to reach an LMO with Eq. (9a).

$${\Delta v}_{LMO}=\mathrm{4,782} \frac{{\text{m}}}{{\text{s}}}$$

The landing on Earth requires around 100 m/s^25^, while the maximum allowable velocity at the periapsis of the hyperbola is 12.5 km/s. According to the previously presented equation for the flight from Earth to Mars, the total *Δv* for the return flight can be expressed with the following equation.

10c $${\Delta v}_{M\to E}=1.05\cdot \left(\mathrm{4,782} \frac{{\text{m}}}{{\text{s}}}+{\Delta v}_{M}+{\Delta v}_{E}+100 \frac{{\text{m}}}{{\text{s}}}\right)+400 \frac{{\text{m}}}{{\text{s}}}$$

The analysis for the return flight from Mars to Earth is executed analogously as for the flight from Earth to Mars.

### ISRU analysis

The initial steps of the analysis, see Sections "Starship system and payload masses" and "Trajectory analysis", will provide the consumable mass and propellant mass that has to be recovered from Martian resources applying ISRU. Deriving the data and evaluating the feasibility for the infrastructure on the Martian surface is approached similarly to the system design, as described in Sections "Starship system and payload masses". The following steps are undertaken:

1. Review of state-of-the-art systems as data basis,
2. Extrapolating mass and power properties for the required ISRU systems,
3. Extrapolating mass for the power systems on the Martian surface,
4. Extrapolating mass for the transportation systems on the Martian surface.

These steps assume that an adaptation of the currently available or developed systems to the needs as present in the outlined mission scenario is possible.

### Evaluation of feasibility

Once all data is compiled by either literature review or calculation, the results are compared to the statements and plans of SpaceX and how they fit. This concerns e.g. mission durations fitting the time needed for ISRU-produced propellant, minimum possible flight times or mission opportunities. It is analyzed if this mission is feasible and if not, which recommendations can be made to improve feasibility.

## Results

### Baseline mission scenario

**Figure 1:**
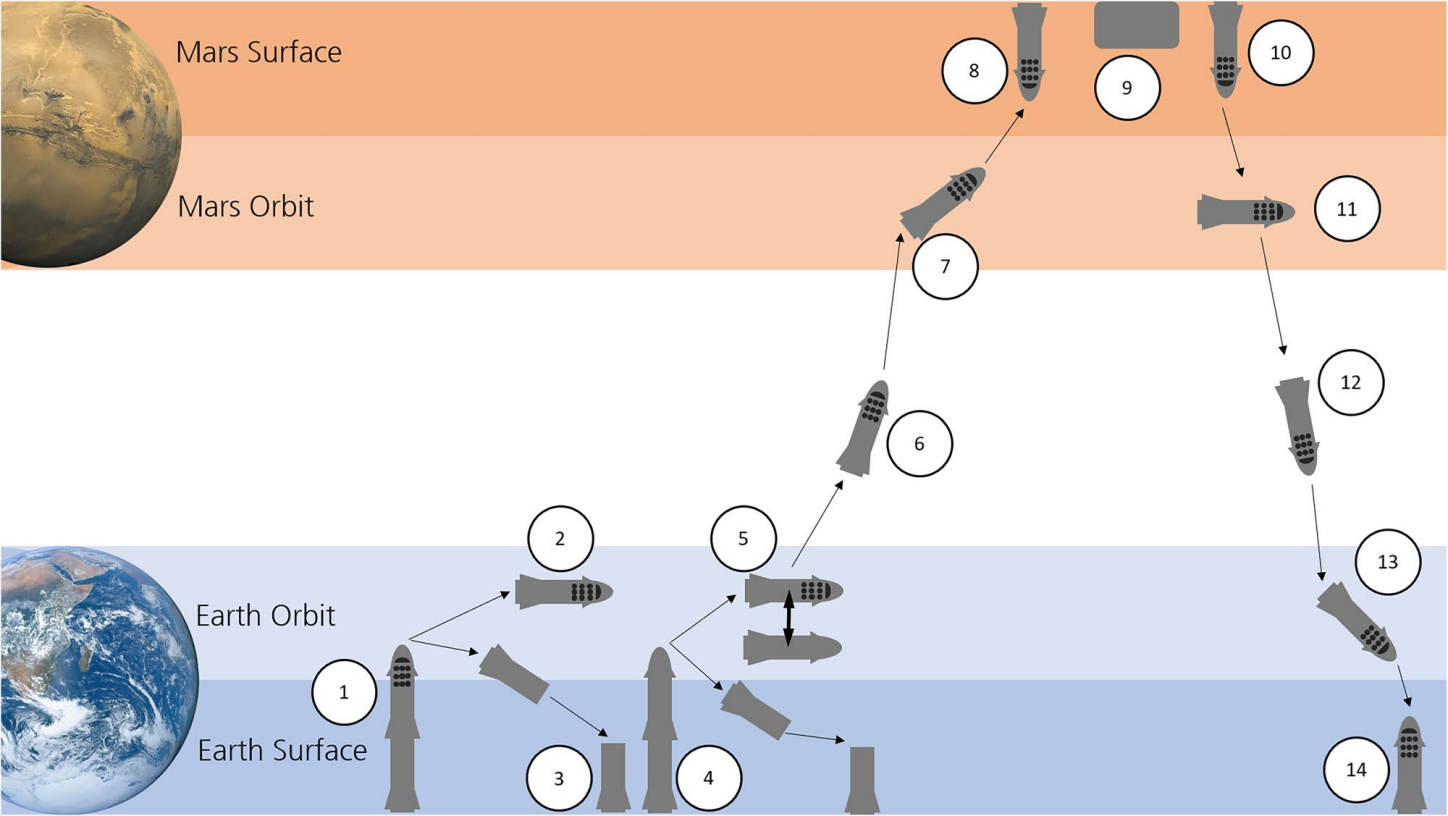
The current baseline scenario for a Mars mission using SpaceX Starship. 1 – Starship launches from Earth. 2 – It reaches LEO, waiting for refueling. 3 – the main stage returns to Earth to be equipped with a cargo version of Starship. 4 – the cargo Starship launches into LEO. 5 – the main stage returns to Earth, while the crewed Starship is refueled. This is repeated until the propellant is sufficient for a Mars mission. 6 – Transfer to Mars. 7 – Aerobraking in Mars atmosphere and 8 – Landing. 9 – During stay on Mars, ISRU is used for propellant generation. 10 – launch from Mars 11 – into a circular orbit and subsequent 12—return to Earth. 13 – Aerobraking is used for 14 – landing on Earth. [Source: Mars and Earth images: NASA, public domain, overall image: own, with information based on^7^].

The baseline scenario for the mission as intended by SpaceX is given in Fig. 1, which is based on^7^. For our purpose we assume two uncrewed missions carrying equipment, e.g. for power generation and ISRU, will launch from Earth in 2027 and two uncrewed and two crewed Starships will travel to Mars in 2029^32,33^, similar to the initial concept^7^, but with a postponed time frame. Starship will launch (1) from Earth and stay in LEO (2), while the main stage returns to Earth (3) and is reused for launching a cargo version of Starship, which subsequently refuels (5) the crewed vessel. This is repeated until sufficient propellant is on board. Starship transfers to Mars (6), where it uses aerobraking in Mars’ atmosphere (7) to reduce its velocity for landing (8). During the stay, ISRU technology produces propellant (9) until Starship launches again (10) into a Mars orbit (11). A transfer orbit injection burn sends Starship on its way to Earth (12), where again aerobraking is used (13) to accomplish landing (14).

SpaceX does not provide information about e.g. orbit altitudes; therefore, we assume a 500 km (altitude) circular orbit for (2). This way, there is sufficient time for refueling, even in case of some launch failure for the subsequent launches, without risking decay of orbit into a realm where Starship can no longer stay on orbit. Also, this is above the ISS, i.e. the risk of collision is reduced. Overall, this orbit altitude has almost no effect on e.g. Δ*v* and therefore can be set arbitrarily. The altitude at Mars at arrival is not fixed, but determined by the maximum possible velocity at closest approach, which is 7.5 km/s according to SpaceX^7^. For the return flight, an initial orbit altitude at Mars (11) is assumed to be 200 km. The approach at Earth (13) occurs at 12.5 km/s maximum [^12^, p. 38], but may not go below 500 km orbit altitude to avoid collision with ISS. As a baseline, the crewed version is assumed to carry 12 persons, but it will also be reviewed for the effect of carrying 100 persons [^8^, p. 5].

For further calculations regarding the mass budget, the following nominal mission values are assumed, based on this given mission scenario. These assumptions are are: ToF of 180 d for flight to Mars and back to Earth, as well as 500 d of surface time. Actual times might differ in the trajectory analysis, but these are assumed as baseline. The ascent to Earth orbit is not regarded as refueling means that the actual mission from a budget point of view starts in LEO.

### Starship mass budget

In the following, the mass budget of Starship as derived within this work is explained. It is based on existing information where available and extrapolated for the remaining values. The goal is to determine a plausible mass budget for the Starship system and subsequently compare it to the proposed values by SpaceX, resp. determine its fit for the mission scenario given by SpaceX.

#### Starship system mass

Starship can carry a payload mass of 100 MT into LEO^34^. A detailed mass budget for Starship itself has not been published by SpaceX. Based on public statements, SpaceX targets at a system dry mass of 100 MT, which includes all subsystems^11^. Assuming a 20% system margin according to ESA standards^13^, this means there are 83.333 MT of mass available for actual subsystems. Of these 4.167 MT are harness, when setting that mass as 5% of the system dry mass without margin, following the same standard^13^. While other numbers have been published in the past, SpaceX gives the propellant mass as 1200 MT on its website^31^. Being the most recent number, this is taken as baseline. Of these, 2% are assumed to be residuals, i.e. not available for actual maneuvers, as stated by ESA standard^13^. Therefore, 1176.47 MT of propellant are available for orbit maneuvers. A summary of these values is given in Table 5 for reference.

**Table 5:** Mass budget baseline for Starship, derived from SpaceX plans and using margin standards by ESA. Indented lines indicated that the upper parameter includes the indented parameter.

Mass parameter	Value	Source
Payload mass	**100 MT**	^34^
System mass w/margin (20%)	**100 MT**	^11^
System mass w/o system margin	83.333 MT	Own calculation, based on^13^
Harness mass	4.167 MT	Own calculation, based on^13^
Propellant mass	**1200 MT**	^31^
Available propellant mass (w/o 2% residuals)	1176.47 MT	Own calculation, based on^13^
**Total launch mass**	**1,400 MT**	Summed up

Significant values are in bold.

In the following an estimate for the subsystems is set up, based on information given by SpaceX where possible or extrapolated from other information, mostly about Orion (see following paragraphs for details), and calculations where necessary. Subsequently, a mass budget is determined and compared to the budget in Table 5.

##### Protection and structure

To minimize the radiation and risk exposure of the crew on a long duration mission to Mars, different protection measures have to be included in the spacecraft. Materials protective against cosmic and solar radiation are e.g. water, polyethylene and aluminium, whereby elements with hydrogen, such as the first two, have a particularly protective effect for both types of radiation^35^. The importance of crew sleeping compartments and control centre leads to the assumption of a polyethylene cover. Furthermore, it is assumed that water pipes (e.g. for water supply and waste water transport) cover as much habitable volume as possible. To minimize the necessary mass, on-board equipment and cargo, e.g. food, are used for radiation protection as well. In the event of a solar flare, similarly to Orion^36^, cargo and food can be used for shelter. Further it was mentioned by SpaceX too that a “central … solar storm shelter^17^” would be provided for the crew. Details were not given.

The habitable volume of the Orion capsule is 9 m^3^ and the total pressurized volume is 20 m^3^^37^. For Starship’s first missions with a crew of twelve, 16% reduction for elements not scaling linearly (e.g. 4 people need one toilet, 12 need not 3 toilets) are assumed, i.e. ten times the volume of Orion for larger cabins and rooms are assumed. Thus, for the model approximately 90 m^3^ habitable and 200 m^3^ total pressurized volume are assumed. The pressurized volume of ISS is 1005 m^3^ for comparison^38^. With a usable diameter of the payload section of 8 m [^8^, p. 2] and thus a base area of about 50 m^2^, the pressurised area is 4 m high, which corresponds to about two habitable floors. The surface area of this cylinder is consequently calculated to:

11 $${S}_{pressurized \,volume}=2\cdot \pi \cdot r\cdot h+1cdot \pi \cdot {r}^{2}=\left(100+1\cdot 50\right) {{\text{m}}}^{2}=150 {{\text{m}}}^{2}$$

It is assumed that the area specific mass of the polyethylene layer is 20 g/cm^2^ (200 kg/m^2^) with a thickness of 0.217 m [^39^, p. 28]. The mass of this shielding is therefore 30 MT. Note only one top side is assumed to be needed to be covered, as the lower side is covered by spacecraft systems and thus is already shielded.

Woolford & Bond report on the habitable volume necessary for human spaceflight missions, which is a function of mission duration, but reaches a plateau at about six to seven months^40^. They provide a so-called performance limit, which is needed if the crew is supposed to conduct tasks and activities, which go beyond survival and also an optimal range. For mission durations of 3 months, the optimum is about 15.5 m^3^, the performance limit is about 7 m^3^^40^. For six months, the values are 20 m^3^ resp. 11.3 m^3^^40^. For 12 crew members, this means, the minimum volume for a 90-day mission is 84 m^3^, the optimal is 186 m^3^. For 180-day missions, which is a realistic flight time at least for some missions, see Section "Trajectory analysis", the values are 135.6 m^3^ resp. 240 m^3^. The assumed 90 m^3^ of this paper thus on the lower range and from a mass budget point of view on the optimistic side. In turn, SpaceX reported previously that they expect a pressurized volume of 825 m^3^ for “40 cabins^17^”. A crew size was not given, but with 40 cabins would exceed the here assumed 12 person crew, i.e. the 825 m^3^ are not regarded.

For micro-meteoroid protection, Starship, similar to the Columbus module of the ISS, is assumed to have a protective layer reinforced with Kevlar and Nextel, a so-called Stuffed Whipple Shield (SWS), which bursts incoming objects with three layers of protective material and thus prevents them from penetrating^41^.

**Figure 2:**
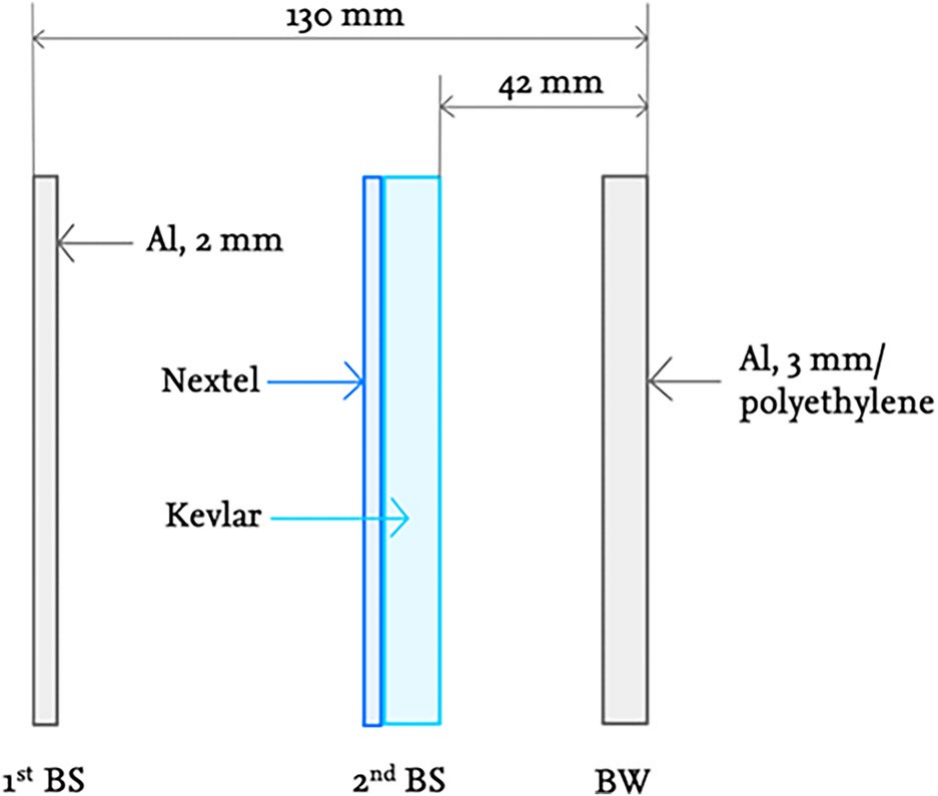
Stuffed Whipple Shield for Starship with two bumper shields (BS) and one back wall (BW), after^41^**.**

The three layers consist of two bumper shields (BS) and the back wall (BW). Since Starship, unlike the Columbus module, will only be in space and on Mars for approximately 2.5 years, the values are oriented to those of the module but have been reduced. For example, the outer layer of the SWS should consist of a 2 mm thick Al 6061-T6 aluminium layer with an areal density of 0.6 g/cm^2^ and the intermediate stuffing of two layers of Nextel 312 AF-62 with 0.2 g/cm^2^ as well as eight layers of Kevlar 129 Style 812 with 0.4 g/cm^2^^41^. On the outer walls of the crewed Sect. (100 m^2^, see Eq. (7), the back wall should not consist of an aluminium layer, but instead of the polyethylene layer of the radiation shielding. In this way, mass can be saved. This results in 1.2 g/cm^2^ (12 kg/m^2^) and therefore 1.2 MT for the SWS around the crewed section of Starship. For the remaining part of Starship, 3 mm thick Al 2219-T851 aluminium with 0.8 g/cm^2^ is to be used as the back wall^41^. For simplification, a height of 40 m is assumed without protection of the engine area, which results in an outer skin of 1005 m^2^ with the same base area of 50 m^2^ according to Eq. (7). With an areal density for this protection of 2 g/cm^2^ (20 kg/m^2^), it results in a mass of 20.1 MT, adding 10% margin, this leads to 22.1 MT. Figure 2 shows the described structure of the SWS for Starship. The dimensions refer to the aluminium and not the polyethylene layer with a thickness of 0.217 m of the crewed section, as this is considerably thicker. However, the distances between the individual layers should be identical.

Furthermore, Starship must be designed and built in such a way that its structure can carry the payload of up to 100 MT with empty tanks, because they will be almost empty by the time it arrives on Mars. To estimate the mass of the remaining structure, the simplification is made that Starship is a 50 m high cylinder with a diameter of 9 m and thus, similarly to Eq. (10c), a surface area of 1541 m^2^. Since this shape is larger than the one of Starship, additional structural elements within the fuselage are compensated for. As with the current prototypes, 3 mm thick 304L stainless steel is used for Starship’s outer skin^42^, which has a density of 8000 kg/m^3^^43^. For the calculation of the outer skin, the areal density is needed, which is the density multiplied by the thickness of the material and thus amounts to 24 kg/m^2^ for the stainless steel used. This results in a mass of 37 MT. With a 10% margin, e.g. for internal structure elements, the structural mass is estimated at 40.7 MT.

For the thermal protection Pica-X is used^44^. It has a density of 0.27 g/cm^3^ and typically has a thickness of 6 cm in a heat shield^44^. Assuming a cylinder of 9 m diameter and 48 m height^17^, as Starship’s size (not regarding the conic nature of its upper part, due to lack of measurement data for that), this yields a surface area of 1357.2 m^2^. Covering that with 6 cm of PICA-X heat shield would mean a volume of 81.43 million cm^3^. With the given density, this would result in a mass for the thermal protection of 22 MT. Assuming not every part needs to be covered with the full 6 cm, but on average 3 cm, would result in 11 MT for the heat shield.

##### Environmental control and life-support system (ECLSS), accommodation and thermal protection

The life-support system, accommodation and thermal control is not provided for Starship by official sources. For Orion, a mass of 1.2 MT is given as mass for these subsystems^18^. It is assumed that these scale with the crew size, e.g. as the amount of CO_2_ produced by the crew is one driver for the ECLSS and that scales with the crew size. Thus, for this calculation this leads to a mass of 3.6 MT (12-person crew, instead of 4-person crew). This is a rough estimate as certain mission parameters are different, e.g. mission duration. Since the value given in^18^ is an estimate as well, no further margin is added here. The Orion ECLSS is also the basis for the ECLSS system of the Lunar Gateway’s Habitation and Logistics Outpost (HALO) module^45^. Mera et al.^45^ state that the operation of the ECLSS for longer mission durations than 30 days concern e.g. the exercise mode and removal of trace contaminants, but indicate that no substantial system change is needed for that. Indications for scaling the system to larger crews and volume are not provided in the paper, so that we remain with the conservative estimate given above.

For thermal insulation, Multi-Layer Insulation (MLI foil) is assumed, which provides additional low radiation shielding. The MLI foil encloses the entire Starship except for the engine bay and the entire crew area. The 40 m high cylinder with a surface area of 1005 m^2^ already mentioned is therefore used as an assumption for the volume to be enclosed, to which the floor and ceiling of the crewed area with 50 m^2^ each are added. The surface area to be covered is thus 1105 m^2^. Good insulation is to be provided by 40 layers of MLI with a surface density of 0.2 g/cm^2^ (2 kg/m^2^)^41^. The mass of the required MLI is thus 2.21 MT.

For additional protection against strong solar storms, special vests are to be available on-board Starship, which should be worn when a solar flare occurs. One such vest is the AstroRad vest, which will be tested on the Artemis missions. The mass of a vest depends on the size of the person wearing it. On average, it weighs 27 kg^46^, which corresponds to a mass of 324 kg for a crew of twelve. Furthermore, the ECLSS is to be expanded to include a radiation warning system that will warn the crew when solar storms occur and they have to seek shelter. The HERA (Hybrid Electronic Radiation Assessor) radiation warning system, which is used on board the Orion capsule, will be used for this purpose^36^.

##### Comms/avionics

For communication and avionics, a similar system as for Orion is assumed, lacking further references and information. The mission profile is similar, although not identical, therefore, the system is not scaled up. For instance, an increased crew size would not necessarily lead to an increase in communication data to be sent or commands to be handled by the system. Therefore, the value for Orion is selected, i.e. 0.6 MT^18^. Again, as this is already an estimated value, no further margin is added.

It has to be noted that the currently intended mission profiles for Orion (lunar environment) and this analysed Mars mission, differs in solar distance, which affects the link budget of the communication system. Considering Mars’ distance of about 1.5 AU and that of Earth of about 1 AU, this means maximum distance would be about 2.5 AU, i.e. resulting in a signal strength of about 1/6 (~ 1/d^2^). This change can be compensated by directiveness of antenna, antenna size, increase in transmitter power or accepting a reduced amount of transmitted data. Especially during transfer, where no significant scientific activities are to be assumed, this change in the link budget does not warrant a larger system. In a Mars environment, communication satellites could also be used as relays for Earth communication, allowing a similar system without further losses. More detailed information about Orion’s communication system is not available, but NASA press releases explain that the current Orion communication system is intended for use beyond the lunar environment^47^.

##### Power

Solar arrays, which are stowed in the engine area during launch and landing and are deployed during the flight, are responsible for the power generation during the flight. Therefore, they must not only be deployable but also retractable. Similar to the Orion capsule, the solar arrays are supposed to have a mechanism that allows them to constantly align themselves with the sun so that they can deliver full power.

Orion’s four 7 m long and 2 m wide solar arrays, each consisting of three foldable panels, provide 11.2 kW of power for a crew of four people^48^. Therefore, Starship’s solar arrays should have about ten times the power, 100 kW. In addition, the radiation intensity decreases by about half during the flight to Mars. In order for the solar arrays to deliver the required power near Mars, they need to deliver at least twice as much power near Earth. With some margin for failing solar cells, for example, an output of around 250 kW is required near the Earth. One solar panel that should be able to deliver this amount of power is the MegaFlex from Northrop Grumman, which is foldable and unfolds into a round panel by rotating 360°. The MegaFlex is a scalable system that is currently still being tested, but its smaller version—the UltraFlex—is already being used on, for example, the Cygnus spacecraft and the InSight lander on Mars^49^. So, the technology is already proven and has a flight heritage. A system consisting of two MegaFlex arrays, each with a diameter of around 24 m, should be able to deliver this power^49,50^. Together, the two arrays have a mass of about 2 MT^49^. To this a 5% margin is added, as the system is already developed.

As with Orion, lithium-ion batteries are to be used to store surplus energy. They have a high energy density and can power Starship in the absence of sunlight and as a back-up^51^. SpaceX could use batteries from Tesla here. It is assumed that the batteries have to provide power over a time span of 6 h in case of a power loss which results with a power of 100 kW in a required battery size of 600 kWh. The 6 h are assumed as no public figure provides information about duration of assumed emergencies. For redundancy there should be second a battery pack with the same size. With the use of the 100-kWh battery from Tesla, which has a mass of 625 kg^52^, and a factor of 1.2 for aging and recharging this results in a mass of 9 MT for the batteries in total. Here as well, a 5% margin is assumed.

The assumed total mass of the EPS, including the solar arrays and a margin of 10% for additional components (e.g. cables), is approximately 12 MT.

##### Propulsion

The propulsion system is based on 6 Raptor engines, each with a mass of 2 MT^10^. It is also using a cryogenic propellant tank, which has to house 1200 MT of propellant^31^. Super Heavy, i.e. the main stage for Starship’s ascent from Earth, has a tank for 3600 MT of propellant with a mass of 80 MT^10^. As there are no further details on the tank system, it must be assumed that the masses given already include the systems for cryogenic propellant storage. Assuming SpaceX will use the same technology for the tank in Starship, the following estimate is made.

The tank mass ${m}_{T}$ can be expressed as:

12 $${m}_{T}={S}_{T}\cdot {d}_{T}\cdot {\rho }_{T}$$

where ${S}_{T}$ is the tank’s surface, ${d}_{T}$ the tank’s wall thickness and ${\rho }_{T}$ the material density. It is assumed that the material and thus density of both tanks (Super Heavy and Starship) are identical. Furthermore, it is assumed that the inside pressure and loads (e.g. during launch) to be withheld are similar as well, i.e. the wall thickness is also assumed to be identical for both tank types. Therefore, for our calculations is true, that:

13 $${m}_{T} \sim {S}_{T}$$

Assuming a spherical tank and using formulas for sphere volume ($=4/3 \cdot \pi \cdot {r}^{3}$) and surface ($=4 \cdot \pi \cdot {r}^{2}$), one can write for the relations between the two:

14 $$\frac{S}{V}=\frac{3}{r}$$

15 $$S=\frac{3}{r}\cdot V$$

16 $$V=\frac{S\cdot r}{3}$$

Considering the propellant mass of 1/3 in comparison to Super Heavy, the Volume of the tanks is regarded as:

17 $${V}_{S}=\frac{1}{3}\cdot {V}_{SH}$$

where the index *S* denominates Starship and *SH* Super Heavy. From this relation one can derive that:

18 $$r_{S}^{3} = \frac{1}{3} \cdot r_{SH}^{3} \Rightarrow r_{S} = \sqrt[3]{1/3} \cdot r_{SH}$$

Using Eqs. (14) and (15), this leads to:

19 $$S_{S} = \frac{1}{{\sqrt[3]{1/3} \cdot r_{SH} }} V_{1} = \frac{{S_{1} }}{{3 \sqrt[3]{1/3}}} = 0.231 \cdot S_{1}$$

With Eq. (12) follows:

20 $${m}_{T,S}=0.231\cdot {m}_{T,SH}=18.49 {\text{MT}}$$

Using the ESA margin for to be modified components, i.e. 10%^13^, this leads to a tank mass for Starship of 20.3 MT. The Helium tanks for the cold gas reaction thrusters^10^ are assumed as 5 MT, this is an estimate as a suitable reference is not available. For the reaction control system (RCS) it is assumed, that 50 RCS thrusters are used for Starship, since the smaller Space Shuttle had 44^53^. There should be two pairs of five thrusters in the front and rear on each side of the flaps, five thrusters in the front in flight direction and five thrusters in the rear against flight direction (aligned like the main thrusters). As a rough estimate for the mass of a thruster, the 220 N RCS thruster of the Orion capsule is used, which has a mass of approximately 2 kg^54^. This results in a mass of approximately 100 kg for Starship’s RCS thrusters. With the 10% margin this results in 5.5 MT for the helium tanks and 0.11 MT for the thrusters respectively. As the raptor engines are mostly developed, only a 5% margin is assumed^13^. This subsystem also requires piping, which is included in the numbers for harness (see Table 5).

#### Crew and consumables

To support a crew of 12 astronauts on their long duration trip to mars, different crew and consumable elements need to be considered. The final crew and payload mass depend highly on the number of astronauts and the time of flight. Therefore, an overview of required masses per astronaut and per astronaut-day is established and shown in Table 6.

**Table 6:** Mass budget baseline assumed for Starship’s crew, payload and consumables per astronaut and day.

Element	Value	Unit	Recovery
Astronaut	80	kg	–
Spacesuit inside	30	kg	–
Spacesuit EVA	60	kg	–
Crew preference items	10	kg/p	–
Crew operational items	5	kg/p	–
Crew safety items	10	kg/p	–
Crew accommodation items	5	kg/p	–
**Total mass**	**200**	**kg/p**	–
Scientific payload	100	kg	–
**Total mass**	**100**	**kg**	–
Oxygen	0.84	kg/p-d	95%
Food (hydrated)	2.4	kg/p-d	0%
Potable water	2.5	kg/p-d	90%
Hygiene water	1.0	kg/p-d	85%
Hygiene items	0.5	kg/p-d	0%
Clothing	0.5	kg/p-d	0%
**Total mass**	**7.74**	**kg/p-d**	–

Significant values are in bold.

As no detailed information on crew and consumable masses are provided by SpaceX, the mass values for the listed elements are selected based on literature research^18,55–57^. The compared values often contain a large scale of deviations depending on the given assumptions. The selected values in Table 6 are assumed to be suitable to establish a first mass model of the described mars mission scenario but may be subject to change. The improvement of life support technologies towards a closed loop system is an important step in realizing long term interplanetary missions. As SpaceX has not yet published any detailed information about the type and quality of recovery systems, that will be used on their mission to mars, a best-case rate of 100% recovery for gases, fluids and solids is assumed to establish a reference mass.

The total consumable mass per person per day m_consumables_ can be calculated using the given recovery factor k_rec_ from Table 6 in formula (21).

21 $${m}_{consumables}={(1-k}_{rec, oxygen})*{m}_{oxygen}+{(1-k}_{rec,food})*{m}_{food}+(1-{k}_{rec,pot,water})*{m}_{pot.water}+{(1-k}_{rec hyg.water})*{m}_{hyg,water}+{(1-k}_{rec,hyg.items})*{m}_{hyg.items}+(1-{k}_{rec,clothing})*{m}_{clothing}$$

A recovery rate of 100% means, that in theory the systems are able to use an initial payload mass required for 12 astronauts for one day and completely recover it. Therefore, the system is by calculation able to supply the crew without any additional storage or resupply for the entire mission duration. The consumable mass m_consumables_ per person per day turns to zero.

The calculation of the crew and consumable mass on a mission with a closed loop ECLSS System can be derived using Eqs. (21) and (22) and are given in Table 7.

**Table 7:** Mass model for a nominal mission duration and recovery of 100%.

Element		Value	Unit
n_astronaut_	Number of astronauts	12	–
TOF_outbound_	Time of flight outbound	180	d
TOF_Surface_	Time of flight surface	500	d
TOF_inbound_	Time of flight inbound	180	d
m_astronaut_	Mass per astronaut	200	kg
m_consumables,initial_	Initial consumable mass per astronaut	7.74	kg/p
m_consumables_	Mass per astronaut day (with recovery)	0	kg/p-d
k_safety_	Safety margin	20	%
m_c&c,outbound_	Mass crew and consumables outbound	3112	kg
m_c&c,surface_	Mass crew and consumables Surface	111	kg
m_c&c,inbound_	Mass crew and consumables inbound	3112	kg

22 $${m}_{c\&c,IB/OB}=\left(1+{k}_{safety}\right)*({n}_{astronaut}*{m}_{astronaut}+{m}_{science}+{m}_{consumables,initial}*{n}_{astronauts}+{n}_{astronaut}*TOF*{m}_{consumables})$$

23 $${m}_{c\&c,surface}=\left(1+{k}_{safety}\right)*({m}_{consumables,initial}*{n}_{astronauts}+{n}_{astronaut}*TOF*{m}_{consumables})$$

While the astronaut masses and the mass of the scientific payload are relevant for the transfer trips, they can be neglected during the surface stay. Here, only the plain consumable masses are relevant to examine the necessary resupply capacities. In the given equations k_safety_ represents the safety factor, n_astronauts_ represents the number of astronauts, m_astronaut_ represents the mass assumed per astronaut (200 kg according to Table 6), m_science_ represents the mass of the scientific payload (100 kg according to Table 6), TOF represents the Time of Flight in days and m_consumables_ represents the mass of consumables required per person per day. As m_consumables_ turns to zero for a recovery rate of 100% the total required consumable mass is not dependent on the ToF anymore.

#### Summary

With the bottom up estimates as formulated in the previous sections a mass budget summary can be formulated. This is shown in Table 8. The total on orbit mass adds to 1510.5 MT, of which 1200 MT are propellant and 100 MT payload and the 12 person crew and their consumables for an ToF of 180 d. This is assuming that 100% of consumables can be recovered by the ECLSS of Starship for the flight. Overall, the total mass on orbit is exceeding the proposed mass summary by SpaceX by more than 100 MT. This is summarized in Table 9 and input for the trajectory calculations in the following section.

**Table 8:** Summary of the estimated bottom up mass budget of a crewed Starship.

Subsystem/element		Mass [MT]	Sum per subsystem [MT]	Ratio subsystem of dry mass w/harness
Structure	Radiation shielding	30	103.8	61%
	Meteoride shielding	22.1		
	Structure	40.7		
	Heat shield	11		
Environment (life-support, thermal control and crew accomodation)	Life support, thermal control, accomodation	3.6	6.1	3.6%
	Radiation vests	0.3		
	MLI	2.2		
Communication/avionics		0.6	0.6	0.4%
Power	Solar arrays	2.1	13.6	8%
	Batteries	9.5		
	Other	1		
Propulsion	Main engines	12.1	38	22.3%
	RCS thrusters	0.1		
	Main tanks	20.3		
	Helium tanks	5.5		
Total dry mass (w/o harness)			162.1	
Harness (5% of total dry mass w/o harness)			8.1	5%
Total dry mass (w/harness, 5%)			**170.2**	
Margin (20% of dry mass)	–	**–**	**34**	
Payload mass	Crew and Consumables	6.3	**106.3**	
	Other Payload	100		
Propellant	**–**	1200	**1200**	
**Total on orbit mass**	**–**	**–**	**1510.5**	

Rounded on the first digit. The ratios (last column) contain rounding errors, leading to a sum not equal to exactly 100%

Significant values are in bold.

**Table 9:** Mass budget comparison for SpaceX Starship as provided by SpaceX (see Table 5) and as derived by estimates in this work (see Table 8).

Mass parameter	Value by SpaceX	Value by estimate in this work
Payload mass	100 MT	100 MT
System mass w/margin (20%)	100 MT	204.2 MT
Propellant mass	1200 MT	1200 MT
Total launch mass	1400 MT	1510.5

### Trajectory analysis

The usable propellant mass is 1176.47 MT (see Section "Starship system mass") and the specific impulse is 378 s^11^. The ratio of launch mass ${m}_{0}$ (the sum of propellant mass, system mass and payload mass) to dry mass ${m}_{d}$ (the launch mass minus the propellant available for orbit maneuvers) is:

24 $$\frac{{m}_{0}}{{m}_{d}}=\frac{1200+204.2+6.3+100}{\left(1200-1176.47\right)+204.2+6.3+100}=4.516$$

The maximum attainable Δ*v* with one fully fueled Starship thus follows, using the rocket equation^27^, to:

25 $${\Delta v}_{max}={I}_{\mathit{sp}}\cdot {g}_{0}\cdot {\text{ln}}\left(\frac{{m}_{0}}{{m}_{d}}\right)=\mathrm{5,588} {\text{m}}/{\text{s}}$$

Any trajectory requiring more Δ*v* than that cannot be flown by Starship during its Mars mission with the baseline Starship design as given in Section "Starship system mass". Without the 2% of propellant left as residuals in the tanks, the mass ratio would actually be 4.865 and ${\Delta v}_{max}$ would become 5864 m/s. Imperfect propellant use leads to losses of more than 275 m/s in Δ*v*.

#### Results in nominal configuration

Due to the varying alignment of the two planets, the needed *Δv* is changing over the course of a 15-year cycle. In general, a transfer becomes feasible every 22 months, an event that is called launch opportunity. Such launch opportunities stay open for 45 to 160 days in the case of Starship. Each launch opportunity was examined with respect to three performance parameters:

- The local minimum *Δv* for which a transfer becomes possible with a maximum time of flight of 180 days and a payload mass of 100 MT
- The local minimum time of flight for which a transfer becomes possible without exceeding the maximum obtainable *Δv* value of 5588 m/s and a payload mass of 100 MT
- The maximum payload mass that can be brought to the Martian surface according to Eq. (6)

**Figure 3:**
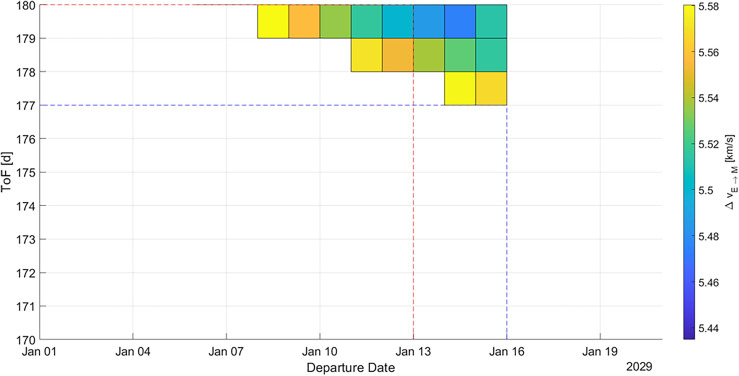
Porkchop plot for an Earth-Mars-transfer in 2028 and 2029. The blue dashed line indicates the minimum ToF trajectory, the red dashed line indicates the minimum *Δv* (and hence maximum payload mass) trajectory. Darker areas indicate lower *Δv* values, bright areas indicate higher *Δv* values and white areas indicate impractical trajectories.

The first analyzed launch opportunity is the one in late 2028 and early 2029, hence the one chosen by SpaceX to have their first manned flight to Mars. We also analyzed the 2033 and 2035 launch opportunities as they show a good performance of the selected three parameters. The results for each launch opportunity are displayed using porkchop plots which show the value of ${\Delta v}_{E\to M}$ for a given tuple of departure date and time of flight. Figure 3 shows the porkchop plot for a transfer from Earth to Mars in 2028 and 2029.

**Figure 4:**
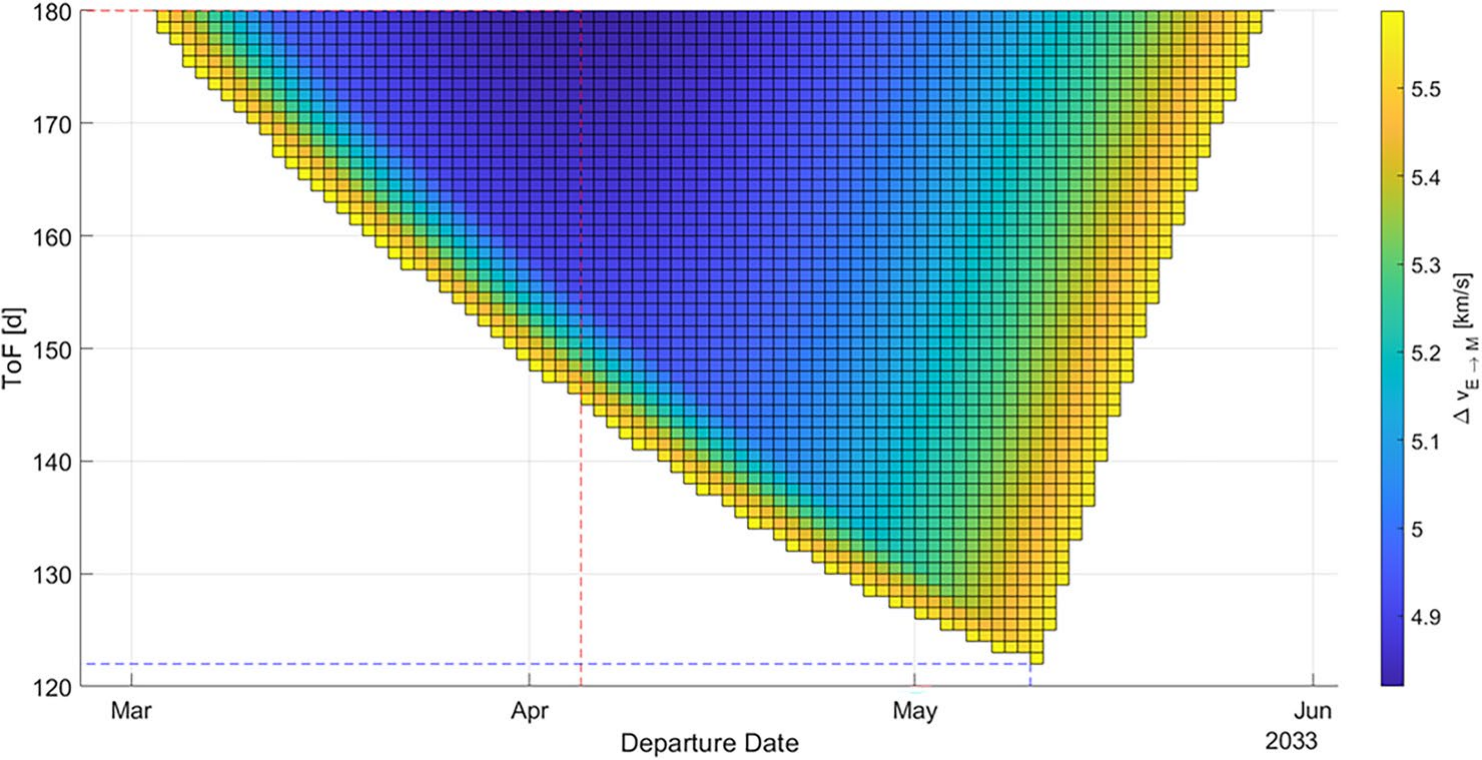
Porkchop plot for an Earth-Mars-transfer in 2033. The blue dashed line indicates the minimum ToF trajectory, the red dashed line indicates the minimum *Δv* (and hence maximum payload mass) trajectory. Darker areas indicate lower *Δv* values, bright areas indicate higher *Δv* values and white areas indicate impractical trajectories.

For that launch opportunity, the minimum *Δv* value is 5435 m/s, corresponding with a maximum payload mass that can be brought to Mars of 114.4 MT. This performance can be achieved with a transfer on 13.01.2029. The minimum possible time of flight in this launch opportunity is 177 d, possible with a transfer on 27.01.2029. In Fig. 4, the porkchop plot for a transfer in 2033 is displayed.

For that launch opportunity, the minimum *Δv* value is 4820 m/s (− 11.3% compared to 2029), corresponding with a maximum payload mass that can be brought to Mars of 178.7 MT (+ 56.2% compared to 2029). Both values are the global minimum/maximum values in the observed time frame. The minimum possible time of flight in this launch opportunity is 122 d.

**Figure 5:**
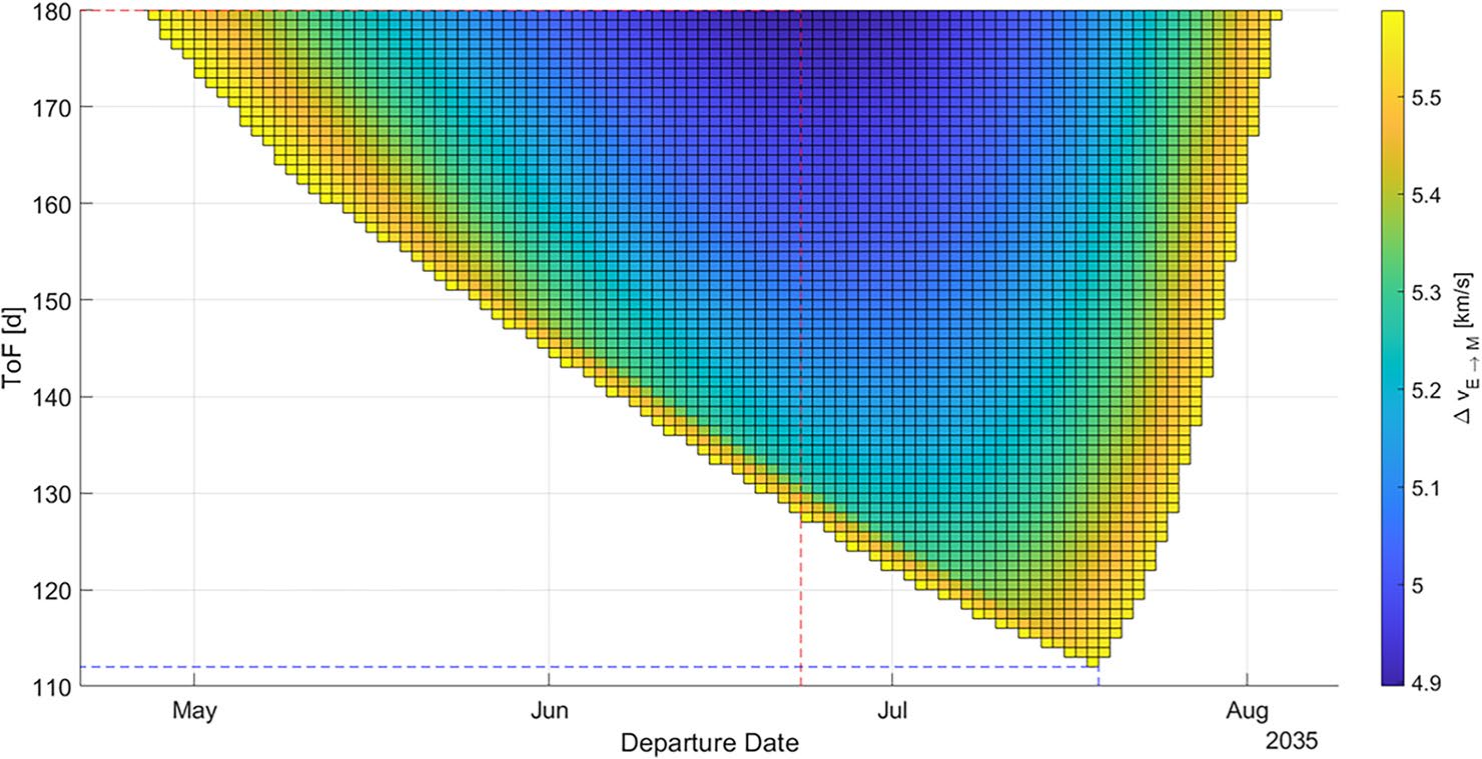
Porkchop plot for an Earth-Mars-transfer in 2035. The blue dashed line indicates the minimum ToF trajectory, the red dashed line indicates the minimum *Δv* (and hence maximum payload mass) trajectory. Darker areas indicate lower *Δv* value, bright areas indicate higher *Δv* values and white areas indicate impractical trajectories.

In Fig. 5, the porkchop plot for a transfer in 2035 is displayed. For that launch opportunity, the minimum is Δ*v* 4896 m/s, corresponding with a maximum payload mass that can be brought to Mars of 170.2 MT. The minimum possible time of flight in this launch opportunity is 112 d (− 36.7% compared to 2029).

#### Sensitivity analysis

Since the results of the previous analysis indicate that the system mass of Starship is likely to exceed 100 MT, it is evident that this is a limiting factor on the performance of the system. The system mass influences the left-hand side of Eq. (6) and therefore the capacity of the system. As a result, the maximum payload mass decreases for higher system masses and the minimum time of flight increases. In order to model the *Δv* required for landing correctly, the structural mass in excess of 100 MT is modeled as additional payload mass. This allows to calculate the maximum payload mass in the same way as in the previous section. Since our analysis showed that the system mass of Starship could exceed the 100 MT as proposed by SpaceX, the following sensitivity analysis examines the advantages of a reduced system mass in terms of mission analysis. We analyzed a transfer in 2033. In Table 10, the *Δv* capacities for a system mass of 175 MT and 150 MT, respectively, are displayed.

**Table 10:** *Δv* capacity for a system mass diverting from the nominal case of 210.5 MT.

System mass [MT]	Δv-capacity
150.0	6176 m/s
175.0	5916 m/s
210.5	5588 m/s

In Table 11, the performance of Starship for the reduced system masses is shown. The performance is measured based on the maximum payload mass and the minimum time of flight. Also, the improvement of the two parameters when compared to our baseline scenario is displayed.

**Table 11:** Starship performance for reduced system Mass concerning possible payload mass and min. flight times.

System mass [MT]	Max. P/L mass [MT]	Improvement	min. ToF [d]	Improvement
150.0	239.2	33.9%	112	8.2%
175.0	214.2	19.9%	115	5.7%
210.5	178.7	–	122	–

It is shown that a reduction of the system mass has only a small influence on the minimum time of flight, but a big impact on the maximum payload mass. These results show the large potential of Starship when reducing the system mass and explain the aims of SpaceX in terms of mission analysis.

#### Feasibility of return flights

According to the presented model in Section "Starship mass budget", return flights from Mars to Earth have been analyzed. The launch opportunities for the return flights were chosen to open 500 days after the landing on Mars, according to the mission plans presented.

in previous sections. Under the assumption that no payload apart from the astronauts and consumables is returned to Earth, the maximum *Δv* for the return flight is 6651 m/s. It has been shown that the ascent to LMO alone consumes 4782 m/s, which are 72% of the *Δv* budget, including margins. Another 6% are used for the TCM, while the landing requires around 2% of the budget. This leaves only 1330 m/s, or 20%, of the maximum *Δv* available for the two remaining maneuvers. In order to set the boundary conditions for the return flight, a maximum time of flight must be chosen. Due to the alignment of the two planets, flight times over 300 d result in a vast increase of required *Δv*. Therefore, we selected 300 d as the maximum allowable time of flight for the return. Before further evaluating the return flight in this configuration, an excursion is needed: If Starship would have a system mass of 100 MT, as proposed by SpaceX, the maximum *Δv* would be 8711 m/s. In this configuration, the global minimum *Δv* for return would be 7121 m/s.

Upon comparison of these two numbers, it becomes evident that a return from Mars to Earth is beyond the capacity of Starship in the presented configuration, since the global minimum for only 100 MT of system mass is already exceeding the actual maximum *Δv* available by about 470m/s.

### In-situ resource utilization

Section "Trajectory analysis" gives an overview of the required propellant masses for different mass- and trajectory options. The results show, that Starship requires the maximum available amount of 1200 MT of propellant on the outbound as well as the inbound trip for the realization of a realistic mission scenario. Following this analysis, it becomes visible, that realizing the described mission to mars with the Starship vehicle is only possible by refilling the spacecraft during the mission.

With a mixture ratio of O/F = 3.6:1^12^ 940 MT of liquid oxygen and 260 MT of liquid methane need to be resupplied as propellant for the inbound trip. In addition, following the calculation in Section "Crew and consumables", the mission requires the resupply of consumable items to support the crew during the surface stay and the inbound trip. The individual as well as total masses can be derived using Eqs. (25) and (26).

26 $${m}_{item, resupply}={m}_{item}*{n}_{astronauts}*ToF$$

27 $${m}_{consumables,resupply}=\sum {m}_{item,resupply}$$

Thus, for a mission with a surface stay of 500 days and an inbound trip of 180 days, 1,263,158 MT of consumable items need to be resupplied for one Starship with a crew of 12 astronauts. In this analysis it is assumed, that two crewed Starship vehicles will return to Earth while the cargo vehicles remain on Mars.

If the amount of 2,526,316 kg is to be resupplied via cargo missions, 26 Starship cargo vehicles with the currently planned payload capacity of 100 MT are required. A reasonable alternative is the production of selected items via ISRU technologies. A detailed overview of the required resupply masses is presented in Table 12.

**Table 12:** Resupply masses for ISRU production.

Element	1 Starship	2 Starships	Unit
m_LOX,resupply_	940,000	1,880,000	kg
m_LCH4,resupply_	260,000	520,000	kg
**m**_**propellant,resupply**_	**1,200,000**	**2,400,000**	**kg**
m_GOX,resupply_	6854	13,708	kg
m_food,resupply_	19,584	39,168	kg
m_pot.water,resupply_	20,400	40,800	kg
m_hyg.water,resupply_	8160	16,320	kg
m_hyg.products,resupply_	4080	8160	kg
m_clothing,resupply_	4080	8160	kg
**m**_**consumables,resupply**_	**63,158**	**126,316**	**kg**
**m**_**resupply,total**_	**1,263,158**	**2,526,316**	**kg**

Significant values are in bold.

#### State of the art

The practice ISRU is becoming quintessential for space exploration as it helps to reduce not only the payload mass and cost, but also cuts down the extra-terrestrial architecture expenditures. In the context of ISRU water and oxygen is the most sought after as it directly correlates to both direct and indirect life support. One such ISRU methodology is the application of membrane technologies for purifying water, minimise wastes and extraction of minerals and useful gases such as oxygen, methane and hydrogen^58,59^. Furthermore, miniature chemical reactors that uses advanced Micro Electro Mechanical Systems (MEMS) and microchannel technology to support the Sabatier process of extracting methane and ethylene production by partial oxidation of methane has been recently developed^60,61^. Another ISRU technique developed is the Methane to Aromatics on Mars (METAMARS) system that converts methane produced from carbon dioxide to low hydrogen aromatic fuels. The system comprises two fully functional oxygen-aromatic hydrocarbon production units sized to produce 1 kg of bipropellant per day^62^.

Further advancements in the ISRU facilities led to the design of scalable in-situ cryogen production facility, which facilitates the capture of high-purity cryogenic fluids, thermal isolation of cryogenically cooled stages and reverse-Brayton cycle cryocooler to liquefy and sustain hydrogen storage^63^. The most recent ISRU technology demonstration is the MOXIE (Mars Oxygen ISRU Experiment) on the Perseverance rover of the 2020 Mars mission of NASA. MOXIE’s performance is driven by solid oxide electrolysis (SOXE) of carbon dioxide, to produce oxygen. It has produced oxygen seven times between landing in February 2021 and December 2021. More details on MOXIE can be found from Hecht et al.^64,65^.

A team of student researchers from the British Columbia University developed and tested a Sabatier Reactor prototype. This Sabatier fuel plant consists of multiple methanation catalysts to enable continuous production of methane. Details of materials and methods needed are presented by Zlindra et al.^66^*.* Furthermore, Baldry et al.^67^ explores the possibilities of integrating several individual ISRU system into one using the power-to-x (P2X) concept. This research explores the potential of P2X in achieving key ISRU outputs such as water, oxygen, methane and other buffer gases as outlined in the NASA design reference architecture 5.0 for a Mars mission^68^. Additional, as it is imperative to improve the TRLs of the ISRU technologies, Starr et al.^69^ has provided a comprehensive overview on the state-of-the-art ISRU facilities such as harvesting and freezing carbon dioxide, water extraction and electrolysis for oxygen and hydrogen production, methanation and purification.

A completely integrated propellant production system that has already been tested on Earth under Mars-like conditions is the Integrated Mars In-Situ Propellant Production System (IMISPPS) from Pioneer Astronautics^70^. It has a single reactor that produces both propellants. The system has a production rate of 1 kg/day, weighs 50 kg and requires 700 W of power^70^.

#### Propellant production system (PPS)

In order to produce the two propellants liquid methane (LCH_4_) and liquid oxygen (LOX) on Mars, a propellant production plant is needed as are water and carbon dioxide to produce methane and oxygen. The water is to be extracted from ice deposits located near the landing site just below the Martian surface or from those found on the surface. A suitable landing site with such deposits must be found beforehand, as this is essential for propellant production and thus also for the return flight to Earth.

For the estimation of the propellant production system (PPS), the maximum of 1200 MT of propellant is assumed. However, since two crewed Starships are to fly back, 2400 MT are required. With a duration of 500 days on Mars and a 30-day safety buffer that the propellant should already be completely produced before the return flight, 470 days are available for production, resulting in a required production rate of 5107 kg/day.

Assuming technological progress based on the IMIPPS over the next few years, the production rate of the system is estimated at 2 kg/day with a system mass of 75 kg and an average power demand of 1 kW. Based on the required production rate and therefore multiplied by

$$\frac{5107 \,{\text{kg}}/{\text{day}}}{2 \,{\text{kg}}/{\text{day}}}=\mathrm{2,553.5}$$

This results in a mass of 191.5 MT and a power demand of 2.55 MW. However, additional power and additional mass will be added for the water extraction, because in the IMISPPS the hydrogen for the Sabatier process was supplied from tanks and not extracted in advance^70^. Since no exact data is available for such a system, it is estimated that the mass and power of the water extraction system is one fifth of the propellant system, so that an additional 38.3 MT and 510 kW are added, giving a total mass of 230 MT and an average power demand of 3.06 MW for the PPS. Both are added with a 10% margin as “to be modified” element, resulting in 253 MT of mass with margin and 3.37 MW of average power demand with margin.

In addition, LOX and LCH_4_ tanks must be built for storage, whereby the tanks of the landed Starships are to be used for this during the first missions. Based on SpaceX’s mission plans (see Section "Baseline mission scenario"), there should already be four uncrewed Starships on Mars ready for LCH_4_ and LOX storage when the first two crewed Starships arrive in 2029. Propellants can be loaded and unloaded via the ports that allow for orbital refuelling. For the transfer of propellants from the Starships converted into storage facilities to the crewed Starships that are to return to Earth, flexible transport pipes must be laid or refuellable rovers used. To prevent the pipes from becoming too long and to keep the distances as short as possible, the Starships must all land close to each other, which is possible thanks to the precise control system. The risk of damage from kicked-up dust and stones should be investigated beforehand.

#### Power supply system

A power supply system (PSS) is needed for propellant production, Starships, rovers, future habitats and all other activities on Mars. Nuclear reactors are to be used as the primary power source, because the use of a solar system as the main power source comes with some disadvantages such as the reduced received energy output on Mars, due to the further distance from the sun. Furthermore, the panels can also only provide power during the day which would be critical during months of dust storms. Dust also accumulates on the panels over time, which also reduces the amount of generate power. Nuclear reactors operate independently of ambient conditions.

Assuming two Starships for the first crewed mission, each requiring 100 kW of power (see Section "Starship system mass"), plus 3.37 MW for the propellant production plant and additional power for e.g. rovers, it is assumed that the PSS must provide 3.6 MW of power. A larger version of the scalable Fission Surface Power (FSP) system should be used as nuclear reactors. Two 2 MW systems, which are required for this power demand, will weigh approximately 32 MT each^71^. This results in a mass for the PSS of 64 MT (since the values used to extrapolate this mass already include margins, no further margin is added here). The stated masses of the systems already include the mass of a protective shield. Furthermore, it must be ensured that in the event of a launch failure, the reactor remains switched off and will not be activated. Since the power already include margins and have been used for scaling, no further margin is included for the mass values.

#### Transportation system

For transport on the Martian surface a transportation system consisting of different modular rovers is needed to transport astronauts and objects and to build infrastructure. To facilitate the construction of infrastructure, rovers are needed that can move the heavy and bulky payloads from the Starships on the Martian soil. For an estimation the mass of around 1000 kg^72^ from the two Mars rovers Curiosity and Perseverance is used. Unlike these rovers, the rovers used should be powered by batteries instead of with Multi-Mission Radioisotope Thermoelectric Generators (MMRTGs), as batteries have a greater availability. Without scientific instruments, but with batteries it is assumed that one rover will have a mass of 800 kg. Five such rovers are to be transported. Including additional modules that can be attached to the rovers for different tasks, a total mass of this subsystem of 10 MT is assumed. Adding a 10% margin, this leads to 11 MT.

#### Summary of surface systems

The total mass of the surface systems is listed in Table 13. A total of 328 MT of mass are estimated to ensure the propellant production for the case of 1200 MT of propellant each for two Starships. No further data by SpaceX was available to the knowledge of the authors during execution of the work described in this paper. These are rough estimates. In addition, 1263 MT of consumables must be delivered or produced via ISRU.

**Table 13:** Mass budget for the ISRU components and misc. equipment on the Martian surface.

System component	Mass estimate [MT]
Propellant production system	253
Power supply system	64
Transportation system	11
Total systems mass	328

## Discussion

### Plausibility of mission scenario and assumptions

The following subsections discuss the different aspects of feasibility, especially concerning plausibility of the assumptions made within this work, but also of the scenario as presented by SpaceX.

#### Starship design

Human spaceflight missions with a long duration conducted with a vehicle and reaching beyond LEO have not been conducted yet, therefore references that are specific and fitting for comparison are not available. For instance, the Space Transportation System was limited to 14-day missions in LEO and the ISS is regularly resupplied, i.e. both are not a good analogue for extrapolating missing data. The closest fit to the operational conditions of Starship is the Orion Multipurpose Crew Vehicle (MPCV), previously the Crew Exploration Vehicle (CEV), which has been designed for mission durations of 21 days^31^ and to enable lunar and Martian missions^18^.

Data about this vehicle is scarce as well, although it has conducted two test missions by now with the Exploration Flight Test-1 in 2014 and with the recent Artemis 1. NASA has published some information about the mass budget^18^ of the vehicle, which can be used as a basis to estimate the plausibility of Starship design. Orion consists of two parts, the service module and the crew module, which together form the overall spacecraft, providing all capabilities. In difference to Starship, Orion will rely on further elements for longer durations and maneuvers exceeding its inherent Δ*v* capability.

A scenario for a Mars mission using Orion has not yet been established, therefore, the given configurations of Orion and of Starship as assumed in this work, based on SpaceX information and scenario, are not fully compatible. Yet, being the only reference, this comparison can provide some estimate on the plausibility still. Exploration missions beyond LEO are conducted with 4 persons for lunar missions. For Mars missions, the mass and crew size are not defined yet in case of Orion.

Comparing both, Starship and Orion as assumed in this work, it can be seen that Orion’s structure mass is considerably lower than for Starship, which is mostly structure mass (61%, see Table 8). In our model for Starship, the Structure subsystem also includes what is labeled as “Protection” and “Other” (e.g. all hatches, inner structure, docking), i.e. the comparison has to occur with those added together, i.e. 43.82% (see Table 2). While the estimate for Starship is factor 1.39 larger than for Orion, one has to consider that Starship also includes structure for landing, which Orion does not. Orion does include structure for docking between Service Module and Crew Module, but Starship also needs an interface for refueling, which would add structure mass. Furthermore, Starship’s heat shielding is supposed to last for several landings, in difference to Orion, which only needs to last for a single landing. The larger ratio of the structure mass to dry mass could also mean that other mass estimates are actually too low, i.e. the mass estimates are in favor of Starship. This is especially true for the ECLSS part, which for a 100% recovery (or near that) rate would likely not be scalable with Orion-based data as done in this estimate, because Orion does not have such a recovery rate. It can be assumed that such an advanced system would result in far more mass. In fact, once more mass data would be available, a trade-off would need to be conducted to determine which option (take more consumables or advanced ECLSS) requires the least mass for the selected ToF. Other comparisons are difficult to make, as a number of systems have been modelled as an extrapolation of Orion values, lacking precise data by SpaceX.

It is however evident that in the assumed best case, manifesting in e.g. an assumed 100% recovery rate of consumables during one mission leg, the mass budget is not fitting the published SpaceX plans as summarized in Table 5. Even in the best-case scenario assumed, the mass budget exceeds the plans by about 100 MT, which is approx. 50% of the mass given by SpaceX.

Less recovery rate would lead to far more mass required for consumables as is closer examined in section "Crew and consumables". Aside from the non-fitting mass budget, this would severely limit the launch opportunities from a trajectory point of view.

A problem for future missions with a crew size of 100 people is the power supply. The power of 100 kW already required for Starship with a crew of twelve, or 250 kW near Earth, would have to be between 2 and 2.5 MW for such a large crew. Solar panels that could deliver such power would probably have to be 60–80 m in diameter if a pair of two 40 m panels is to produce 700 kW and with a slightly exponential power-to-size ratio^49^. Such large panels not only entail the difficulty that they have to be retractable, but also that they are relatively long, estimated at 20–30 m when folded, and have to be stowed on or in Starship. No solar arrays can be seen in current renderings of Starship, only in the very first design of the ITS (Interplanetary Transportation System). This possibly indicates that SpaceX itself is moving away from solar arrays and wants to rely on nuclear reactors such as the FSP system. One of the advantages of these is that the system does not have to be designed to be twice as powerful near Earth in order to deliver the required power near Mars. But then there would be the problem of mass, which is estimated at around 20 t for a 1 MW system (following^71^), and the question of the compatibility of a nuclear power supply and people on board a spacecraft. Another alternative could be to rely on solar panels, but attach them after launch as an external module. This however would need to be compatible with Starship’s exhaust and thus would still need to be designed or could cause redesigns of Starship.

#### Crew and consumables

In the presented mass model in Table 7, the best case with a recovery rate of 100% for all types of consumables was taken as baseline, which is beyond the current state of the art concerning life-support systems.

Available systems usually rely on the implementation of partially regenerative physical–chemical Environmental Control and Life Support Systems that are equipped with current state of the art technology. These systems are assumed to be capable to partially recycle gases with a rate of 95% and fluids with a rate of 90% while solids with a rate of 0% fully rely on resupply processes. The recovery rates for these systems are significantly lower than 100% and result in an increase of the overall consumable masses required for the mission that can be calculated according to the equations provided in Section "Crew and consumables". The detailed figures of the applicable crew and consumable masses are depicted in Table 14.

**Table 14:** Mass model for a state-of-the-art recovery systems.

Element		Value	Unit
n_astronaut_	Number of astronauts	12	–
TOF_outbound_	Time of flight outbound	180	d
TOF_Surface_	Time of flight surface	500	d
TOF_inbound_	Time of flight inbound	180	d
k_rec,gases_	Recovery rate gases	95	%
k_rec,fluids_	Recovery rate fluids	90	%
k_rec,solids_	Recovery rate solids	0	%
m_astronaut_	Mass per astronaut	200	kg
m_consumables_	Mass per astronaut day (with recovery)	3.79	kg/p-d
k_safety_	Safety margin	20	%
m_c&c,outbound_	Mass crew and consumables outbound	12,935	kg
m_c&c,surface_	Mass crew and consumables Surface	27,399	kg
m_c&c,inbound_	Mass crew and consumables inbound	12,935	kg

In case of contingencies on the outbound trip to mars, free return trajectories offer the opportunity to remain on the elliptical transfer orbit for a duration of maximum 3 years and return to Earth without the need for additional maneuvers. Especially for early crewed missions to the red planet this option serves as a relevant safety option. The increased ToF in this scenario leads to a significant increase of the required consumable masses. The crew and consumable masses required to support a crew of 12 astronauts on a contingency trajectory of 1095 days using a state-of-the-art ECLSS system with assumed recovery rates of 95% gases, 90% fluids and 0% solids are depicted in Table 15.

**Table 15:** Mass model for 12 person crew and 3-year free return mission scenario with recovery rates of 95% gases, 90% fluids and 0% solids.

Element		Value	Unit
n_astronaut_	Number of astronauts	12	–
TOF_freereturn_	Time of flight freereturn	1095	d
k_rec,gases_	Recovery rate gases	95	%
k_rec,fluids_	Recovery rate fluids	90	%
k_rec,solids_	Recovery rate solids	0	%
m_astronaut_	Mass per astronaut	200	kg
m_consumables_	Mass per astronaut day (with recovery)	3.79	kg/p-d
k_safety_	Safety margin	20	%
m_c&c,freereturn_	Mass crew and consumables outbound	62,872	kg

It is stated by SpaceX that Starship will be able to transport 100 astronauts to the Martian surface [8, p. 5]. The increased number of astronauts again leads to a significant increase of the required crew and consumable masses. The detailed numbers for a nominal mission without free return option are depicted in Table 16. If a free return trajectory is to be considered for a crew of 100 astronauts, the total required crew and consumable masses multiply by a factor of approximately 5 as listed in Table 17.

**Table 16:** Mass model for a crew of 100 persons on a nominal mission.

Element		Value	Unit
n_astronaut_	Number of astronauts	100	–
TOF_outbound_	Time of flight outbound	180	d
TOF_Surface_	Time of flight surface	500	d
TOF_inbound_	Time of flight inbound	180	d
k_rec,gases_	Recovery rate gases	95	%
k_rec,fluids_	Recovery rate fluids	90	%
k_rec,solids_	Recovery rate solids	0	%
m_astronaut_	Mass per astronaut	200	kg
m_consumables_	Mass per astronaut day (with recovery)	3.79	kg/p-d
k_safety_	Safety margin	20	%
m_c&c,outbound_	Mass crew and consumables outbound	106,913	kg
m_c&c,surface_	Mass crew and consumables Surface	228,329	kg
m_c&c,inbound_	Mass crew and consumables inbound	106,913	kg

**Table 17:** Mass model for a crew of 100 persons with free return option.

Element		Value	Unit
n_astronaut_	Number of astronauts	100	–
TOF_outbound_	Time of flight outbound	1095	d
k_rec,gases_	Recovery rate gases	95	%
k_rec,fluids_	Recovery rate fluids	90	%
k_rec,solids_	Recovery rate solids	0	%
m_astronaut_	Mass per astronaut	200	kg
m_consumables_	Mass per astronaut day (with recovery)	3.79	kg/p-d
k_safety_	Safety margin	20	%
m_c&c,outbound_	Mass crew and consumables outbound	523,055	kg

While the best-case scenario with a recovery rate of 100% delivers the smallest required consumable mass, it is not considered as realistic. During early missions with launch dates in the 2020s and 2030s the use of state-of-the-art-like systems with limited recovery rates seems to be more likely. In addition, these early missions are considered to include a detailed preparation for possible contingency scenarios. Due to these assumptions the included crew and consumable mass should be considered up to ten times higher than the best-case assumption.

For long-term missions beyond the 2030s the availability of optimized systems with significantly improved recovery rates becomes more likely. In addition, technology approval and mission experience offer the chance to reduce the possibility of contingency scenarios. At the same time, long-term missions have the goal to include a larger crew. This might lead to systems that require less mass per person per day and for contingency scenarios. Due to the larger number of crew members that need to be supported, the total amount of required consumables will nevertheless remain high compared to the best-case scenario.

While a higher consumable mass could be compensated by a lower payload mass in early mission scenarios, this option is not reasonable for long-term mission scenarios as the required mass exceeds the included payload capacity of 100 MT. In order to reduce the required consumable masses for all crewed missions to mars, the development of optimized ECLSS Systems with improved recovery rates is a critical task. As the final mass model is highly dependent on the detailed mission layout, the overall mission set-up and mass model including strategies for consumable supply and contingency scenarios implemented by SpaceX is of large interest.

#### Trajectories, launch windows and mission sequence

Section "Trajectory analysis" presents the minimum Δv, the maximum payload mass and the minimum ToF for a given system mass in a selected launch window. While the targeted payload mass of 100 MT can be reached in every launch opportunity, the resulting ToF significantly exceeds the targets communicated by SpaceX. According to SpaceX, their target times of flight between Earth and Mars are 140 d in 2029, 90 d in 2033 and 80 d in 2035^12^, which is the lowest number they are aiming for. As it has been shown in Section "Trajectory analysis", these ToF cannot be reproduced with our model. In fact, these target ToF are missed by over 30 days in every launch opportunity. Still, Starship has shown the capability to bring at least 100 MT of payload to the Martian surface in every launch opportunity.

Given that the targeted scenario by SpaceX seems to be unlikely to be reached according to our analysis, a realistic scenario shall be presented in the following. It seems reasonable that the most important target is to deliver 100 MT of payload to Mars with every flight. This enables the establishment of a Mars settlement, which is a goal of Elon Musk. In order to achieve this, the desired ToF by SpaceX must be increased. For the first flight in 2029, a ToF of 175 d seems reasonable with some slight improvements made until launch, for the 2033 launch opportunity, SpaceX should aim for a ToF of 120 d and for 2035, 110 d seem achievable.

If aiming to reach the lowest ToF as proposed by SpaceX of 80 d, Starship would need to be able to perform *Δv* maneuvers of 8672 m/s, if planning to deliver 100 MT of payload, or 8453 m/s, if planning to deliver no payload. This is well in extend of the current capabilities of Starship. At the IAC 2016, Elon Musk said that he thinks Starship will be able to reach Mars in 30 days in “the more distant future^20^”. In order to reach this goal, Starship would need to be able to perform *Δv* maneuvers of 28,085 m/s, if planning to deliver 100 MT of payload, or 27,866 m/s, without any payload.

For future analysis in this regard, we only consider the case of 100 MT payload and a desired ToF of 80 d (since this is the scenario proposed by SpaceX; Case 1) and the case of 0 payload and a desired ToF of 30 d (Case 2). In order to assess the technical improvements needed to achieve these goals, we first assume that the system mass of Starship can be reduced to 100 MT, as proposed by SpaceX. This would lower the needed *Δv* to 8430 m/s in the first case and 27,689 m/s in the second case.

The rocket equation implies that in this scenario, one can either increase the specific impulse of the engine or the propellant mass to improve the *Δv* capability of Starship. Table 18 shows the required values of these parameters if the respective other remains untouched.

**Table 18:** Changes in either specific impulse or propellant mass to achieve mission scenarios Case 1 and Case 2, if the respective other parameter remains unchanged.

Parameter	Case 1	Case 2
Specific impulse	441.8 s	1100.8 s
Propellant mass	1743.9 MT	175,282.8 MT

Looking first at case 2, it is evident that this propellant mass may not be achieved with any technology known today. Considering that Starship together with Super Heavy is already the biggest rocket ever built, the number seems unreachable, being around 150 times as high as currently. The specific impulse is also high, out of reach for any chemical rocket engine. The highest specific impulse by a chemical rocket engine is 465.5 s, the capability of the Aerojet Rocketdyne RL10 engine used in the Delta III and IV rockets^73^. The impulse of 1100.8 s is more in the range of typical ion thrusters than in the range of chemical engines. But since ion thrusters require the spacecraft to travel on a low-thrust trajectory, the presented trajectory model cannot be used to evaluate this possibility.

When looking at case 1, it becomes evident that the required impulse is lower than the one of the RL10 engine. So, one may argue that this number is not out of reach. What must be considered though, is that RL10 features a LH2/LOX propulsion system. These systems usually reach higher specific impulses than LH_2_ /LCH_4_ systems. The propellant mass in this case marks an increase of about 50%.

In general, there are ways to theoretically reach these values and hence enable transfers in 80 and 30 days. But all these approaches discussed require a significant design change of Starship, which will turn it into a new spacecraft. Concluding it can be said that Starship in its current design does not support these plans.

The sensitivity analysis showed the potential for improving the mission analysis in terms of payload masses and times of flight for a reduced system mass. Considering that neither the propellant mass nor the specific impulse can be improved on a big scale, the system mass is the parameter which can have the greatest influence on the performance of the system. It seems reasonable for SpaceX to focus their improvements on this field.

A special attention should be given to the results presented in Section "Feasibility of return flights", where it has been shown that Starship in the configuration presented in this paper is not capable of flying back to Earth as the required Δ*v* exceeds the possible Δ*v*. This has some important influences on the mission design:

1. The astronauts flying to Mars cannot return.
2. Starship cannot be used as a reusable spacecraft.
3. The mission plans by SpaceX are not feasible in their current form.

Combining the aforementioned aspects, we assess that from a mission analysis point of view, based on the by us extrapolated mission scenario and the limited available information about Starship, the mission scenario and spacecraft capabilities do not fit. Within the boundaries of our analysis it would be required to significantly lower Starship’s system mass.

### Technology readiness

The radiation and micro-meteoroid protection as well as the components of the ECLSS, COMMS and TCS are technologies that have already been used on previous spacecraft and therefore have a high degree of technology readiness. The MegaFlex solar arrays of the EPS are based on the UltraFlex, which has a flight heritage and, with TRL 9, the highest possible technology readiness level (TRL). A MegaFlex array with a diameter of 9.6 m has already been tested in the course of a TRL 5 demonstration, but it has not yet been tested in space or in the size required for Starship. As the technology is available but still needs to be scaled to the required size and a mechanism for retracting the solar arrays needs to be developed, it is quite possible that this could be operational by the planned launch date of 2029.

The next new technology to be considered are Starship’s main engines, the Raptor engines. At the time of writing, they have not yet been used in space, but will be from 2023 onwards during the orbital test flights. However, they have already been used in numerous tests on Earth, so their level of technology readiness is medium (approx. 6). For the RCS thrusters, for which no more precise specifications are yet available, it can be assumed that existing thrusters or similar to these will be used here, so that the technology readiness will also be given for these. The system for orbital refuelling, however, has not yet been developed. The feasibility of such a system must be demonstrated by the launch of the first cargo Starships and successfully tested during several tests, which is considered feasible due to the need to carry out all Mars missions on the scale SpaceX is planning.

The heat shield technology also still needs to be extensively tested during re-entries and possibly adapted, depending on what the tests reveal. However, it is believed that the heat shield tiles will meet their protection and reusability requirements by the first launch in 2027, especially since SpaceX already has experience designing a heat shield for the Dragon space capsule.

That the technology to produce liquid methane and oxygen would work on Mars has already been demonstrated with the IMISPPS. However, this system has only a fraction of the propellant production rate needed to fuel two Starships. In addition, such a system without a water extraction plant requires with 2.55 MW a lot of power even with the assumptions of technological progress that have been made. Therefore, it is seen as critical that such a system is operational and flight-ready by 2029. The current technology readiness and feasibility are therefore low. As the ISRU activities shall enable return of the crew to Earth, their reliability is of upmost importance and needs to be tested and proven in advance of any mission.

The technology readiness of the Fission Surface Power System is also not yet very advanced. Technology demonstration has already taken place successfully on Earth with a smaller 1 kW reactor. The problem with the FSP, however, is the estimated time to operational capability. Systems with 10–20 kW are expected to be flight-ready in 3–5 years, the required 2 MW system only in probably ten years^71^ (source from 2021). It can therefore be assumed that this technology will not be available for the planned 2029 mission.

Table 19 lists the new technologies used and their current TRL status. Not much is known about the current state of research. More funding for research on these topics will certainly help a lot for raising their current TRL.

**Table 19:** TRL status of new technologies used.

Technology	TRL
MegaFlex solar arrays	Medium
Raptor engines	Medium (expected to be high within 2023 or 2024)
Orbital refuelling	Low
Heat shield	Medium
Propellant production system	Low
Fission surface power system	Low

Due to the lack of precise publications on respective TRL, general labels have been used based on published information about tests. *Low* TRL 1–3, *Medium* TRL: 4–6, *High* TRL 7–9 (not used), but expected for Raptor engines.

### Plausibility of ISRU

The payloads to be transported for the first Starships are the propellant production system, the power supply system, the rovers as well as the crew consumables. A system mass of 253 MT was assumed for the PPS. The total mass of the PSS, consisting of two 2 MW systems of 32 MT each, has a total mass of 64 MT. Five rovers of 800 kg each plus additional modules were estimated at 11 MT. For the consumables this scenario would lead to ca. 126 MT of consumables. According to the baseline scenario, a total of four cargo Starships and two crewed Starships with a payload capacity of 100 MT each are available until 2029 to bring the required systems to Mars. Considering the mass alone, 454 MT would have to be distributed among six Starships with a payload capacity of 600 MT. This would be feasible, but it presupposes that the volumes of the payloads can also be distributed appropriately among the Starships as the payload volumes of the Starships are limited. Thus, three cargo Starships could be used purely for the PPS and one cargo Starship purely for the PSS. The rovers could then be transported either in one of these Starships or on board one of the crewed Starships. Since the PPS should ideally remain on board the Starships after landing in order to reduce the logistical effort, but it must be divided among three Starships, the Starships must land very close to each other so that pipes for connecting the individual units are as short as possible. The consumables are to be transported as payload on board the crewed Starships.

Starting the propellant production already two years earlier would drastically reduce the required power of the system. At 1200 days, the production rate of the PPS would have to be only 2000 kg/day, with a mass of 75 MT and 1 MW of power, including the water extraction system, that is about 90 MT and 1.2 MW. However, the feasibility of this idea is difficult because the system, which is distributed over several Starships, would have to be connected by robots to form one system. In addition, the Starships would have to land practically directly on an ice deposit so that it could be used directly for production. In addition, there is the connection of the PSS with the PPS and the transport of the produced propellant into the propellant tanks of the second cargo Starship for storage. All processes would therefore have to be executed automatically by robots, whose control cannot take place in real time either. If anything should go wrong and the system cannot produce any propellant, this can only be fixed when the crew lands two years later and then the propellant production system is designed too small to produce the required amount of propellant for the return flight in the remaining time span (or the mission has to be cancelled). Of course, such a problem can also occur during a crewed mission, but a human being is better able to solve an initially unknown problem. Starting propellant production only with the arrival of the crewed Starships may therefore seem risky at first and also definitely represent a risk factor, but in the end, it is probably the safer way. Moreover, even with the extended production period, a 2 MW reactor is still needed, and its availability of ten years remains unchanged.

### Long-term sustainability of missions

According to SpaceX and Elon Musk, Starship is intended to enable humanity “becoming a multi-planet species^17^”, with long-term settlements on the Martian surface^17^. This can only be the case if mars missions are conducted sustainably. Sustainability is the result – likely never to be achieved but striven for – of sustainable development, which per definition “is the development that meets the needs of the present without compromising the ability of future generations to meet their own needs^74^”. Sustainable development has three dimensions, namely ecology, economy and social^75^.

Achieving sustainability or ensuring sustainable development requires more than e.g. ISRU technology to reduce the dependence on resupply from Earth. Social and ecological aspects have to be addressed, which e.g. concerning the living conditions on Mars, polluting Mars or exploiting resources. Water ice used for fuel generation, is not present for future generations (on Mars) or for a possibly existing ecosystem on Mars – up to now it has not been excluded yet, that Mars holds life of its own.

Governance on Mars, where a settlement has to be more than an outpost of a company. If humanity is to become a multiplanetary society, rules have to be established with special care of not repeating similar mistakes as before during the age of colonization, which resulted in exploitation of resources and humans. Repercussions of that are even reaching into contemporary times still.

Currently frameworks describing sustainability in the context of space missions are lacking a system view, which entails all three dimensions of sustainable development^76^ and do not ensure actual sustainability is attained. Starship does likely reduce the environmental impact of spaceflight, due to its reusability, but more aspects are needed to ensure actual sustainability. This has to be addressed in the scenario’s plans in the future to be fully evaluated.

### Overall feasibility

It has been shown that the currently available information and extrapolation does not lead to a feasible mission scenario as published by SpaceX. Most significantly, even assuming ISRU-technology available, a return flight cannot be achieved with Starship. While a refueling approach as for the flight towards Mars could be an option, this is considered to risky in the hostile environment for Mars, especially, considering that failure would leave the crew stranded. An approach with as little complexity as possible is needed, i.e. significant technology developments are needed.

The biggest problems that have arisen are caused by the PSS, PPS and EPS and concern the mass, their required power and their produced power, respectively, and the technology readiness. With the currently available technology for propellant production, this system requires too much power for the size it needs for production for two Starships and is also too heavy. Technological progress was already assumed in the calculation of the PPS. Should this not occur, the required power and mass would increase by 40% and 30% respectively compared to the assumed values. Distributing the PPS, PSS and rovers among the four cargo and two crewed Starships with a standard payload capacity of 100 MT should be feasible in terms of mass and also volume.

As the main problem however, the high-power requirement of the PPS of 3.37 MW is seen, which leads to heavy nuclear reactors with high power. In addition to the mass, these have the problem that their technology readiness is not yet very high, which in turn leads to high development and construction costs as well as a long-time span until flight readiness of about ten years.

Due to the lack of alternatives for the problematic systems described above, these hurdles cannot be avoided more easily with other technologies. The use of solar panels instead of nuclear reactors represents too great a risk in dust storms, and there is no way around a propellant production system, since transporting 2400 MT of propellant to Mars is also not practical and therefore not feasible. For these reasons, it is concluded that SpaceX’s expanded mission plans in the baseline scenario are not achievable and feasible at this scale and timeframe by 2027/2029.

If the time until the nuclear reactors of the PSS are actually ready for deployment is ten years, this would be deployable in 2031. This would also allow time for the development and scaling of the PPS, which in the best case can be made smaller, lighter and more power-efficient by then. If these hurdles can be overcome by then, the first Starships could be launched in the mid 2030’s. For these launch windows, a feasibility analysis must then be carried out again based on the required velocity changes, the duration of stay on Mars and thus the demands on the PPS.

### Open issues

Not mentioned in this paper before is the need for elevators on Starship, which can be seen in some of SpaceX’s and NASA’s renderings. The fact that an elevator does not yet exist that has to bring astronauts and payloads to the surface of Mars even during dust storms is problematic, as this is something that has not existed before and the requirements for this system are probably very high. This is because the elevators must be able to operate even during dust storms. The moving components, which are then particularly exposed to sand, must therefore be designed in such a way that sand cannot penetrate the system anywhere and lead to malfunctions.

The issue of planetary protection should also be considered in detail in order to keep human contamination of Mars for scientific experiments as low as possible. However, this cannot be completely avoided when astronauts set foot on the surface.

Furthermore, it could be investigated if a refueling in Mars orbit scenario would enable return flights. Such a refueling in Mars orbit scenario is not part of what has been currently found in SpaceX plans. Such refueling would involve autonomous docking or remotely controlled docking with time delays of up to about 40 minutes in Mars orbit, when controlled and coordinated from Earth. Neither has been done before and thus is either a high-risk activity or has to be developed and proven further. Due to that lower readiness and due to the fact that orbital refueling at Mars is not part of the currently available Mars mission scenario, it has not been investigated within this work. Similarly, the level of autonomy of ISRU infrastructure, as well as the interaction of its parts and possesses, assumed or needed for the mission has to be investigated^57,77–79^.

SpaceX has not provided detailed mission plans, especially not concerning contingency scenarios, e.g. if a free return trajectory is to be used or some other form of redundancy concept to ensure the crew’s safety. In a similar regard, details about the assumed ECLSS system, especially concerning recovery rates should be provided.

Another open issue is, if the selected technology influences the landing site, e.g. concerning radiation, temperature, illumination, planetary weather, topography or landing Δ*v*.

### Recommendations

It has been shown that the current plans for Starship Mars missions and their feasibility show significant gaps. For closing these gaps and improving feasibility, the following recommendations are made:

- Aim for uncrewed Mars mission to improve mission reliability and heritage via testing of essential mission elements under Mars conditions
- Include more (international) partners, incl. possibly political organizations (of the space sector or others) to enhance the necessary technology development in relevant fields such as ISRU, Power generation, ECLSS
- The scenario analyzed in depth here has shown that a high recovery rate of consumables is relevant to reduce the mass and even with a 100% recovery at the moment, the mass is not fitting the mission requirements, therefore, developing life-support systems with a large recovery rate is mandatory to not further increase the gap between performance and actual mass
- Possibly use one-way cargo versions of Starship to reduce amount of propellant that has to be created in-situ and use them as stationary elements instead of infrastructure elements, which have to be transported along with the crewed Starships, e.g. for habitation or re-using Starship’s solar panels

## Conclusion

This paper has compiled a feasibility analysis for Starship based on a published mission scenario and extrapolation of existing systems, where information about Starship had gaps. Using typical analysis methods, a mass budget for the system and subsystems was established. A Lambert solver was applied to identify the minimum ToF and Δ*v*. It has been shown that there are currently several gaps in the available technology to conduct a Mars mission as sketched by SpaceX, e.g. concerning ISRU capability, power supply and the performance of Starship itself, which based on the mass estimate presented here, is incapable to conduct the mission as proposed by SpaceX. Especially, the ToF limits published by SpaceX are found to be unrealistic and cannot be held with the current design, requiring at least further improvement of the performance, some are outright physically impossible (i.e. Mars cannot be reached within 30 days with such a transfer vehicle). The current estimate does also not allow the return flight of Starship. Even with an unrealistic 100% recovery rate of consumables, the mission was not feasible for a 12 person crew per Starship, let alone for the SpaceX published 100 person crew. Further technology development is required, to supplement this launch and transfer vehicle and enable Mars missions. This is affecting Starship itself, but also infrastructure elements needed for the SpaceX proposed mission, especially those required for ISRU-based production of propellant. With the information currently available a Mars mission with Starship is not feasible.

## Supplementary Information

Supplementary Information.

## Data Availability

All data generated or analysed during this study are included in this published article and its supplementary data file, containing the plot data used to generate the porkchop plots Figs. 3, 4, 5.

## Abbreviations

CEV: Crew exploration vehicle
ECLSS: Environment and life-support system
ESA: European Space Agency
EPS: Electrical power system
ISRU: In-situ resource utilization
ISS: International Space Station
ITS: International transportation system
LEO: Low-Earth orbit
LMO: Low-Mars orbit
LSS: Life-support system
MAV: Mars Ascent Vehicle
MLI: Multi-layer insulation
MOI: Mars orbit insertion
MPCV: Multi-purpose Crew Vehicle
MT: Metric tons
PPS: Propellant production system
PSS: Power supply system
RCS: Reaction control system
SWS: Stuffed whipple shield
TCM: Trajectory correction maneuvers
ToF: Time of flight
TOI: Transfer orbit injection
TPS: Thermal protection system
TWR: Thrust to weight ratio

## Symbols

*a*: Semi-major axis
*d*: Material thickness
*E*: Excentric anomaly
*g*_0_: Standard gravity acceleration
*I*_sp_: Mass specific impulse
*K*: Recovery factor
*m*: Mass
*µ*: Gravitational parameter
*S*: Surface area
*r*: Radial position/radius
*ρ*: Material density
*t*: Time
*v*: Velocity
*V*: Volume
