# Author Correction: About feasibility of SpaceX's human exploration Mars mission scenario with Starship

**DOI:** 10.1038/s41598-024-71955-6

**Published:** 2024-09-05

**Authors:** Volker Maiwald, Mika Bauerfeind, Svenja Fälker, Bjarne Westphal, Christian Bach

**Affiliations:** 1 German Aerospace Center (DLR), Institute of Space Systems, Bremen, Germany; 2 Faculty of Production Engineering, University of Bremen, Bremen, Germany; 3 Chair of Space Systems, Technical University Dresden, Dresden, Germany; 4 Technical University Braunschweig, Braunschweig, Germany

Correction to: *Scientific Reports* 10.1038/s41598-024-54012-0, published online 23 May 2024

In the original version of this Article, the maximum Δv for the return flight was incorrect.

As a result, in the Results section, under the subheading ‘Feasibility of return flights’,

“Therefore, we selected 300 d as the maximum allowable time of flight for the return. Before further evaluating the return flight in this configuration, an excursion is needed: If Starship would have a system mass of 100 MT, as proposed by SpaceX, the maximum Δv would be 8711 m/s. In this configuration, the global minimum Δv for return would be 7771 m/s.

Upon comparison of these two numbers, it becomes evident that a return from Mars to Earth is beyond the capacity of Starship in the presented configuration, since the global minimum for only 100 MT of system mass is already exceeding the actual maximum Δv available by more than 1100 m/s.”

now reads,

“Therefore, we selected 300 d as the maximum allowable time of flight for the return. Before further evaluating the return flight in this configuration, an excursion is needed: If Starship would have a system mass of 100 MT, as proposed by SpaceX, the maximum Δv would be 8711 m/s. In this configuration, the global minimum Δv for return would be 7121 m/s.

Upon comparison of these two numbers, it becomes evident that a return from Mars to Earth is beyond the capacity of Starship in the presented configuration, since the global minimum for only 100 MT of system mass is already exceeding the actual maximum Δv available by about 470 m/s.”

The original Article has been corrected.

